# RAD-Seq analysis of wild Japanese garlic (*Allium macrostemon* Bunge) growing in Japan revealed that this neglected crop was previously actively utilized

**DOI:** 10.1038/s41598-023-43537-5

**Published:** 2023-09-29

**Authors:** Wiwit Probowati, Shogo Koga, Kentaro Harada, Yukio Nagano, Atsushi J. Nagano, Kanji Ishimaru, Kazusato Ohshima, Shinji Fukuda

**Affiliations:** 1 The United Graduate School of Agricultural Sciences, Kagoshima University, Kagoshima, Japan; 2 Center for Education and Research in Agricultural Innovation, Saga University, Saga, Japan; 3 Faculty of Agriculture, Saga University, Saga, Japan; 4 Analytical Research Center for Experimental Sciences, Saga University, Saga, Japan; 5 Faculty of Agriculture, Ryukoku University, Otsu, Japan; 6 Institute for Advanced Biosciences, Keio University, Tsuruoka, Yamagata Japan

**Keywords:** Agricultural genetics, Population genetics

## Abstract

*Allium macrostemon* Bunge, commonly referred to as "no-biru" in Japan, is a widespread wild onion species found across the country. Despite being deeply entwined in ancient Japanese culture, it remains an underutilized crop in Japan. Determining the origins of its domestic populations and understanding their genetic composition is crucial to highlighting the plant's historical significance in Japan. This study aims to bridge this knowledge gap by examining the genetic diversity of 47 *A. macrostemon* samples from various regions in Japan using RAD-Seq. Our analyses distinguished unique population structures, dividing the samples into three distinct groups: A, B, and C. Notably, groups A and B showed clear evidence of bulb propagation, while group C did not. Group C formed four subgroups: C1, C2, C3, and C4. Hybridization between subgroup C1 and either group A, B, or both, resulted in the emergence of subgroups C2, C3, and C4. Thus, groups A, B, and C1 are posited as the ancestral populations. Additionally, our morphological observations indicated distinct differences among these three groups. Our findings also suggest that human migration may have influenced the plant's distribution, hinting at active usage in the past that later waned, causing its current underutilized status.

## Introduction

Wild Japanese garlic (*Allium macrostemon* Bunge), also known as "Chinese garlic" in English and "no-biru" in Japanese, belongs to the Amaryllidaceae family^1^. The plant is naturally distributed across eastern Russia, eastern Mongolia, mainland China, Taiwan, Korea, and Japan^2–5^. It has been utilized in traditional East Asian medicine for centuries^1, 6, 7^, in addition to serving as a food source. While *A. macrostemon* is notably popular as an edible *Allium* species in Korea and northeastern China^4^, Russia primarily uses its bulbs for pickling^8^. However, Japan's use of this plant deviates from these patterns. It is mostly harvested from the wild and occasionally sold in local markets, with cultivation limited to small scales. Hence, despite its extensive distribution, *A. macrostemon* is currently considered a neglected crop in Japan, possibly due to its low yield leading to underutilization^9^.

Historical references suggest that this plant was used medicinally in Japan^10^. Its mentions in classical Japanese literature point to active utilization in the past. Waka poems referencing this plant appear in the Kojiki ("Records of Ancient Matters"), compiled in 712, and the Manyoshu ("Collection of Ten Thousand Leaves"), an anthology of various waka poems written until 759. One waka poem in the Manyoshu mentions the plant's use in marinated food, while another describes the use of the bulbs for medicinal treatment. Despite these clues indicating the prior widespread use of this neglected crop, supportive genetic evidence is lacking. Therefore, studying the genetic diversity of *A. macrostemon* in Japan could shed light on this enigma.

The plant possesses significant medicinal properties, renowned for its analgesic, immune-boosting, and anti-asthmatic capabilities in traditional East Asian medicine^1, 6, 7^. Recent investigations into the chemical compounds found in *A. macrostemon* have revealed impressive potential for medical applications. Its bulb is rich in steroidal saponins, which show promise in treating conditions like acute myocardial ischemia^11^, hyperglycemia, hyperlipidemia, and visceral obesity^12^. These steroidal saponins also exhibit cytotoxic activities against human cancer cell lines^13^. Moreover, an aqueous extract from the dried bulbs reportedly shows antidepressant-like and analgesic effects^14, 15^. The leaves of *A. macrostemon* contain valuable constituents, such as flavonoids^16^, caffeic acid^17^, and phenolic compounds^18, 19^, suggesting potential utility in herbal medicine. Additionally, the plant's volatile organic compounds possess medicinal properties, including lipid-lowering and anti-atherosclerosis effects^20^. These discoveries underscore the remarkable potential of *A. macrostemon* as a herbal medicine.

Despite numerous studies exploring the medicinal components of *A. macrostemon*, its genetic diversity remains understudied. However, understanding genetic diversity is crucial as it provides a vital tool for monitoring and evaluating populations, which aids in the conservation planning of *A. macrostemon*. Moreover, knowledge of this plant's genetic diversity allows plant breeders to develop and improve new cultivars with desirable traits, such as pest and disease resistance, high yield potential, and superior characteristics^21^.

Compared to traditional DNA markers, single nucleotide polymorphisms (SNPs) are more abundant in plant genomes, making them a superior tool for identifying plant diversity^22^. Advances in high-throughput sequencing technologies have significantly reduced the cost and improved the efficiency of obtaining SNP markers, particularly through restriction site-associated DNA sequencing (RAD-Seq)^23^. This method facilitates the reduced representation of individual genomes. A RAD-Seq variation, termed double-digest RAD-Seq (ddRAD-Seq), utilizes two restriction enzymes to cut the genome^24^. Compared to traditional RAD-Seq methods, ddRAD-Seq library preparation is less costly, quicker, and requires less genomic material^25^. Consequently, ddRAD-Seq has become the preferred choice for species lacking genome information, such as *A. macrostemon*. No reports exist on the genetic relationships and intraspecific diversity of *A. macrostemon* using either traditional or high-throughput methods. Encouraged by our research group's previous successes with RAD-Seq or ddRAD-Seq in analyzing various species, including loquat^26, 27^, Japanese pepper^28^, citrus^29–31^, and firefly^32^, we are inspired to apply this knowledge to the study of *A. macrostemon*.

In this study, we explore the genetic diversity and population structure of wild Japanese garlic, *A. macrostemon*. We collected 47 samples from various regions in Japan, including Honshu, Kyushu, Shikoku, and Okinawa Island, with Hokkaido excluded. Subsequently, we conducted an in-depth analysis using genome-wide SNP markers obtained via RAD-Seq. Our objective is to reveal the historical context of *A. macrostemon* in Japan, particularly its association with past human activities.

## Results

### Sample collection of *Allium macrostemon*

**Figure 1:**
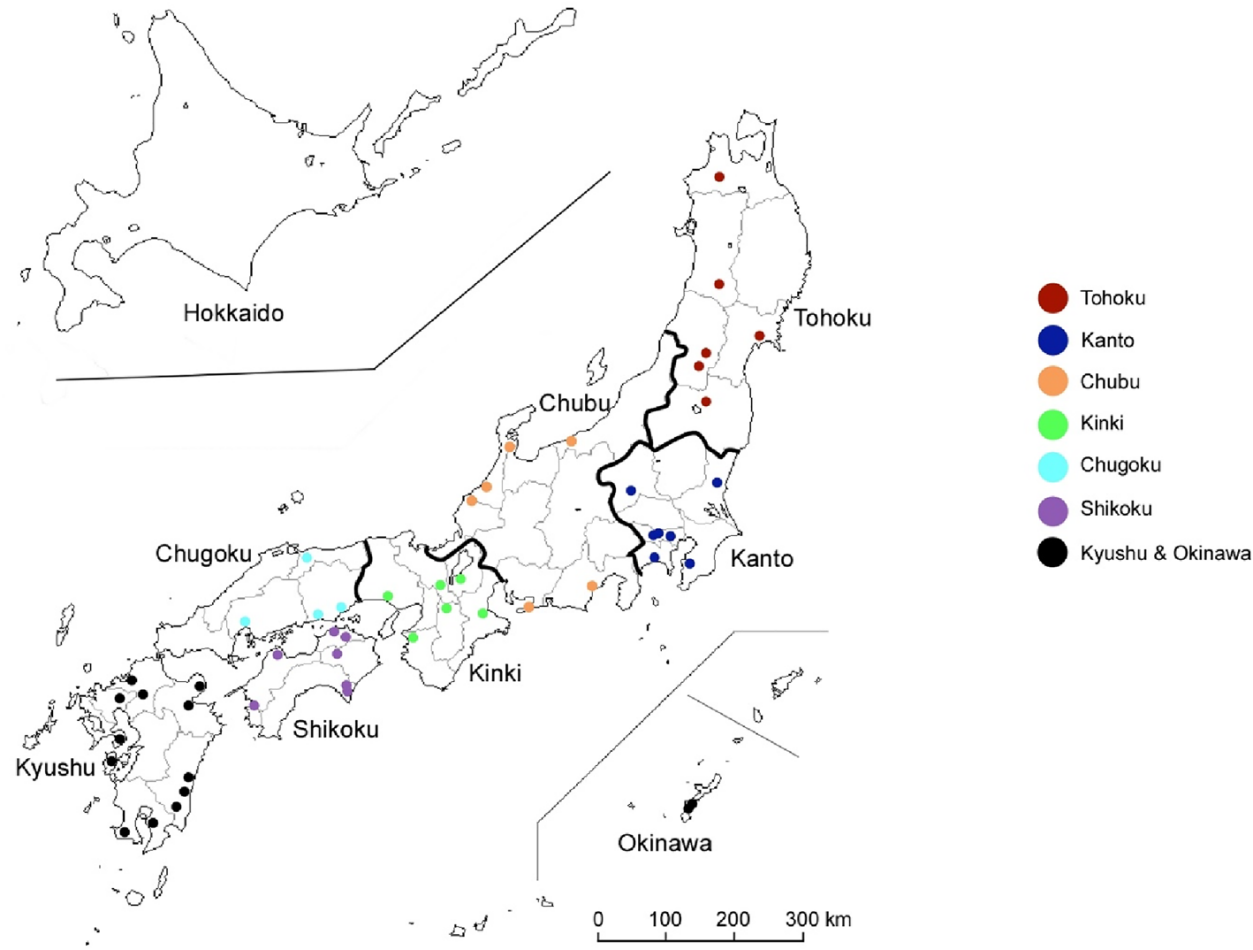
Collection sites for *Allium macrostemon*. Colours are used to refer to the different districts. The map is sourced from http://www.craftmap.box-i.net/ (accessed on 21st February 2023).

We collected bulbs from 47 *A. macrostemon* plants from different locations in Japan, including fields and riverbanks. Figure 1 and Table 1 show details regarding the plants, their collection dates, and places of origin. We further created three technical replicate samples (samples 30 and 31, samples 14 and 15, as well as samples 17 and 32), resulting in a total of 50 samples subjected to ddRAD-Seq.

**Table 1:** Information on *Allium macrostemon* samples used in the study.

No	Isolate	Location (city, district, prefecture)	Collection date	Group
5	WOA360	Inakadate-mura, Minamitsugaru-gun, Aomori	8-Aug-14	Group C1
33	WOAK354	Shincho, Yonezawa, Akita	15-Jun-14	Group C1
22	WOYA331	Shiromori, Yamagata, Yamagata	12-Jun-14	Group C1
10	WOYA335	Kawatoi, Nanyo, Yamagata	12-Jun-14	Group C1
24	WOFK488	Akogashima, Atami-machi, Koriyama, Fukushima	23-Apr-15	Group C2
21	WOMY326	Nishiutari, Hamaichi, Higashimatsushima, Miyagi	13-Jun-14	Group B
2	WOGU466	Shimotoyooka-machi, Takasaki, Gunma	16-Mar-15	Group B
12	WOIB449	Senba-cho, Mito, Ibaraki	15-Mar-15	Group B
30	WOTK389	Utsuki-machi, Hachioji, Tokyo	7-Dec-14	Group B
31	WOTK389_1	Utsuki-machi, Hachioji, Tokyo	7-Dec-14	Group B
48	WOC441	Kamezawa, Futtsu, Chiba	14-Mar-15	Group C1
29	WOKN373	Terasaka, Oiso-machi, Naka-gun, Kanagawa	5-Dec-14	Group C4
25	WOST86	Kawaguchi, Saitama	8-Jul-13	Group B
11	WONI551	Nishinaka, Itoigawa, Niigata	18-Apr-15	Group C2
7	WOTY540	Shiragawa, Himi, Toyama	18-Apr-15	Group C2
23	WOIS532	Shima-machi, Komatsu, Ishikawa	17-Apr-15	Group C2
18	WOFI554	Kumasaka, Awara, Fukui	17-Apr-15	Group C3
49	WOAI220	Gonjochi62, Tahara, Aichi	18-Apr-14	Group C1
8	WOSH405	Echigoshima, Yaizu, Shizuoka	24-Jan-15	Group B
6	WOSG538	Yasu, Shiga	16-Apr-15	Group C4
1	WOKY536	Shimogyo-ku, Kyoto, Kyoto	17-Apr-15	Group C3
47	WOHG425	Shimizuueno, Uozumi-machi, Akasi, Hyogo	7-Mar-15	Group C4
9	WONR546	Denen, Gozyo, Nara	20-Apr-15	Group C1
27	WOME413	Koazakacho, Matsusaka, Mie	25-Jan-15	Group B
13	WOW560	Wakayama, Wakayama	20-Apr-15	Group C4
19	WOT105	Hoki-cho, Saihaku-gun, Tottori	14-Nov-13	Group C4
20	WOOY574	Tamashimakurosaki, Kurashiki, Okayama	9-Mar-15	Group C3
42	WOOY575	Okutyofukutani, Setouchi, Okayama	8-Mar-15	Group A
35	WOHR72	Yahatahigashi, Nakaji, Itsukaichi-cho, Saeki-ku, Hiroshima, Hiroshima	10-Mar-13	Group A
26	WOKW581	Konanchooka, Takamatsu, Kagawa	10-Mar-15	Group A
34	WOKW582	Ayautachookadakami, Marugame, Kagawa	10-Mar-15	Group A
3	WOTS607	Kamo, Higashimiyoshi-cho, Miyoshi-gun, Tokushima	10-Mar-15	Group C3
4	WOEH562	Niya, Imabari, Ehime	13-Mar-15	Group C1
36	WOEH566	Takata, Tsushima-cho, Uwajima, Ehime	13-Mar-15	Group A
14	WOKO594	Sakihama-cho, Muroto, Kochi	12-Mar-15	Group B
15	WOKO594_1	Sakihama-cho, Muroto, Kochi	12-Mar-15	Group B
39	WOF366	Kitano-machi, Kurume, Fukuoka	14-Nov-14	Group A
40	WOF395	Higashi-ku, Fukuoka, Fukuoka	16-Dec-14	Group C4
43	WOS317	Saga, Saga	13-Feb-15	Group C1
28	WOO199	Akimachishimobaru, Kunisaki, Oita	12-Feb-15	Group C4
45	WOO200	Ishigakihigashi, Beppu, Oita	12-Feb-15	Group C3
50	WON227	Shirakami, Unzen, Nagasaki	9-Feb-15	Group C4
41	WOK289	Shimoshima, Amakusa, Kumamoto,	9-Feb-15	Group C3
38	WOM366	Komono, Hanagashima-cho, Miyazaki, Miyazaki	11-Feb-15	Group C4
46	WOM367	Noziri-cho, Kobayashi, Miyazaki	11-Feb-15	Group C3
44	WOM368	Sakataniko, Nichinan, Miyazaki	12-Feb-15	Group C1
37	WOKG194	Hamabira, Tarumizu, Kagoshima	9-Feb-15	Group C3
16	WOKG195	Myoken-machi, Makurazaki, Kagoshima	10-Feb-15	Group C3
17	WOOK122	Chunjun, Kitanakagusukuson, Nakagami-gun, Okinawa	10-Dec-13	Group C4
32	WOOK122_1	Chunjun, Kitanakagusukuson, Nakagami-gun, Okinawa	10-Dec-13	Group C4

### Variants detection using RAD-Seq data

The ddRAD-Seq of the 50 samples, as shown in Table 1, generated more than 10 Gbp of data, comprising a total of 196.2 million raw, single-end 51-bp reads. Quality-based filtering yielded an average of 0.9 million reads (ranging from a minimum of 0.2 million to a maximum of 3 million) across the 50 samples (Supplementary Table S1). The Stacks program-built loci de novo with an average coverage depth of 16.78-fold (Supplementary Table S2). We identified a total of 5848 variant sites for subsequent analysis.

### The plant consists of three groups

**Figure 2:**
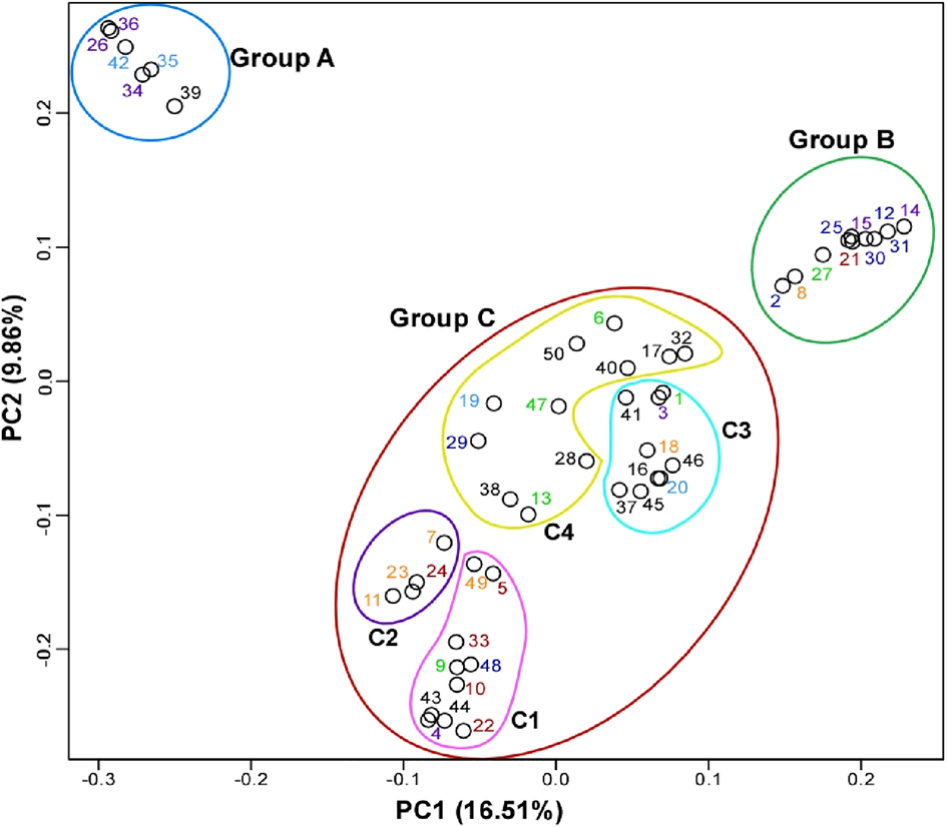
Principal component analysis (PCA) of *Allium macrostemon* samples, with the first two components based on 5848 SNP markers. The contribution rate of each principal component is indicated in parentheses. The colour scheme of the samples corresponds to that in Fig. 1. Colours are used to refer to the different groups. The figure was generated using R software (version 4.1.2).

**Figure 3:**
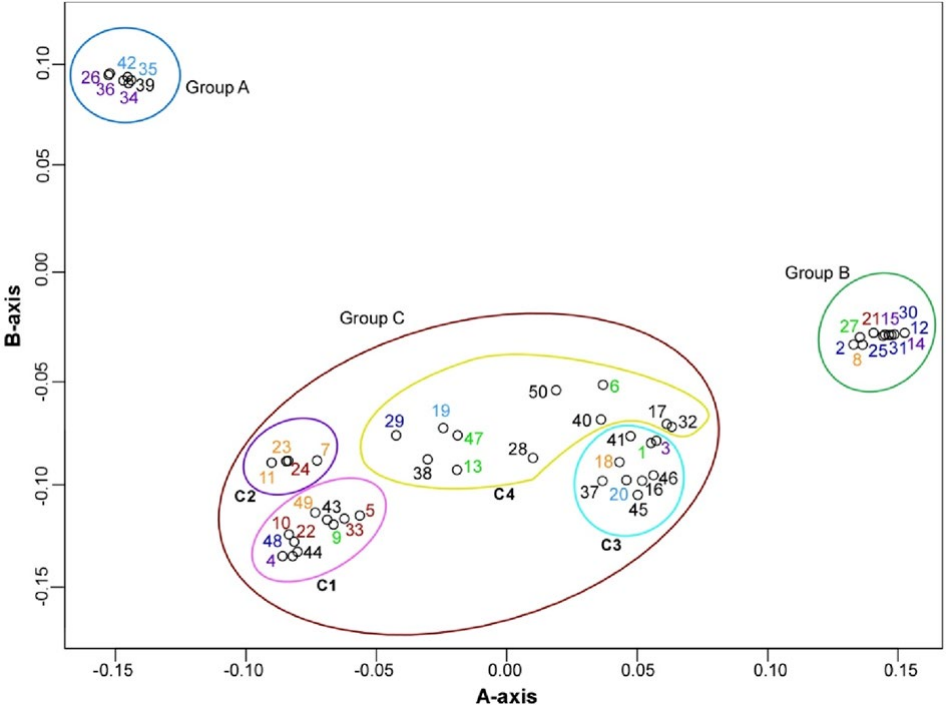
Multidimensional scaling analysis of *Allium macrostemon* samples using two-dimensional data based on 5848 SNP markers. The colour schemes of the samples and the groupings correspond to those in Figs. 1 and 2, respectively. This figure was generated using R software (version 4.1.2).

Principal component analysis (PCA) of these variant sites effectively partitioned the 50 samples into three groups, labeled A, B, and C (Fig. 2). The division into these three groups was primarily driven by the principal components 1 and 2, which contribute 16.5% and 9.86% respectively. Analysis of principal components 3, 4, and 5 did not reveal additional meaningful features (Supplementary Fig. S1). Multidimensional scaling (MDS) analysis classified the samples into three groups, with the same members as identified by the PCA (Fig. 3).

Members of group A originated from three districts in Japan: the plants in Chugoku (35 and 42), Shikoku (26, 34, and 36), and Kyushu (39). Thus, they were found throughout Western Japan, excluding the Okinawa district. Members of group B, on the other hand, came from five districts in Japan: plants from Tohoku (21), Kanto (2, 12, 25, and 30, with 30's technical replicate 31), Chubu (8), Kinki (27), and Shikoku (14, and its technical replicate 15). These were absent from the Chugoku, Kyushu, and Okinawa districts, indicating that they were not found in Western Japan, except for the presence in the Shikoku district. Members of group C were from all districts in Japan: plants in Tohoku (5, 10, 22, 24, and 33), Kanto (29 and 48), Chubu (7, 11, 18, 23, and 49), Kinki (1, 6, 9, 13, and 47), Chugoku (19 and 20), Shikoku (3 and 36), Kyushu (16, 28, 37, 38, 40, 41, 43, 44, 45, 46, and 50), and Okinawa (17 and its technical replicate 32).

**Figure 4:**
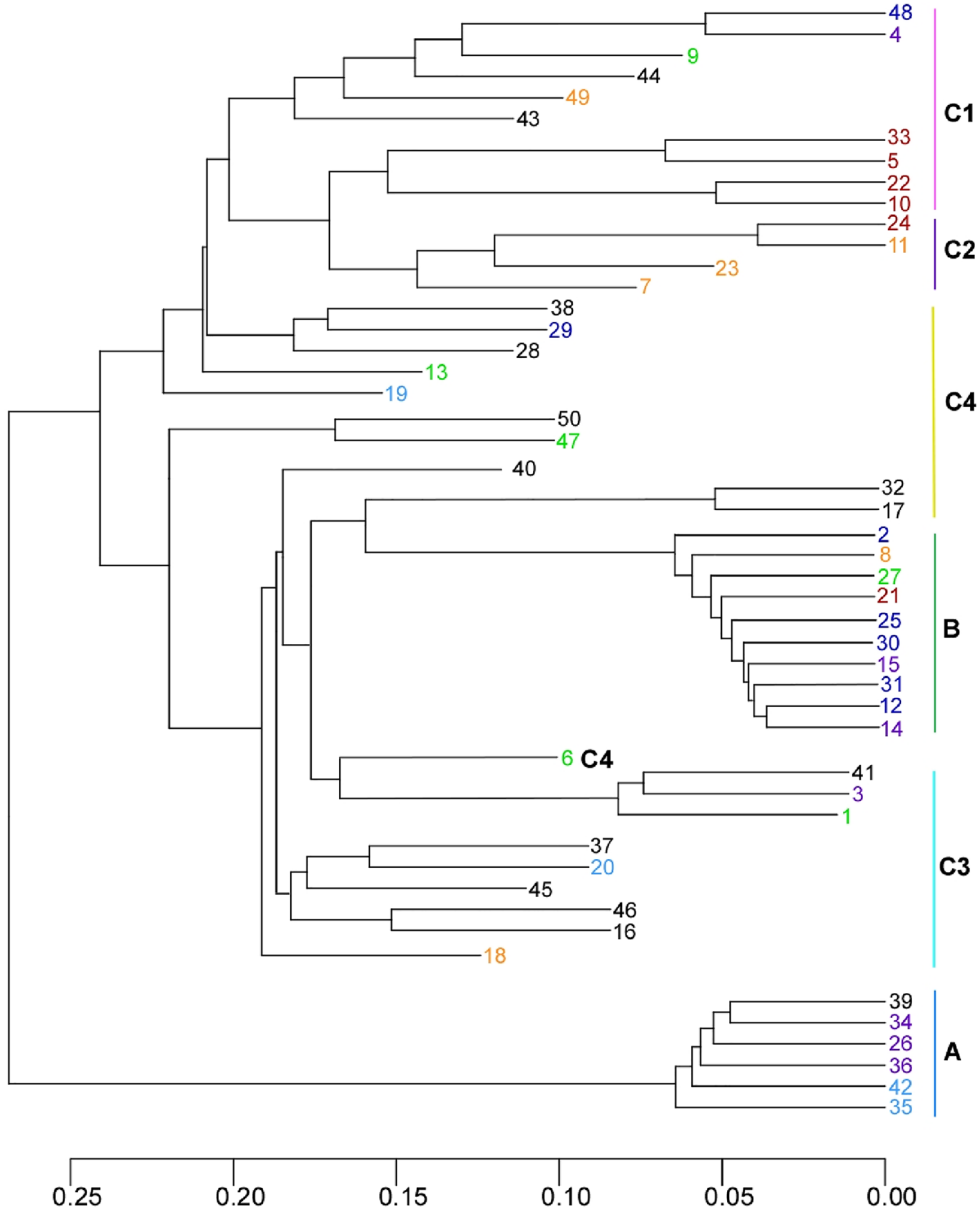
Cluster analysis of *Allium macrostemon* samples. The colour schemes of the samples and the groupings correspond to those in Figs. 1 and 2, respectively. This figure was generated using R software (version 4.1.2).

Cluster analysis was performed using a pairwise distance matrix (Fig. 4), which identified the same three groups as those identified in the PCA and MDS analyses: groups A and B each formed a tight cluster, while group C was an outgroup of groups A and B. The cluster analysis detected several subclusters, but, with two exceptions, it did not detect any region-specific subclusters. The two exceptions are the subclusters to which plants 5, 10, 22, and 33 from the Tohoku district and plants 16 and 46 from the Kyushu district belong.

### Gene flow from group A and/or group B to group C was detected, but not from group C to group A or group B

**Figure 5:**
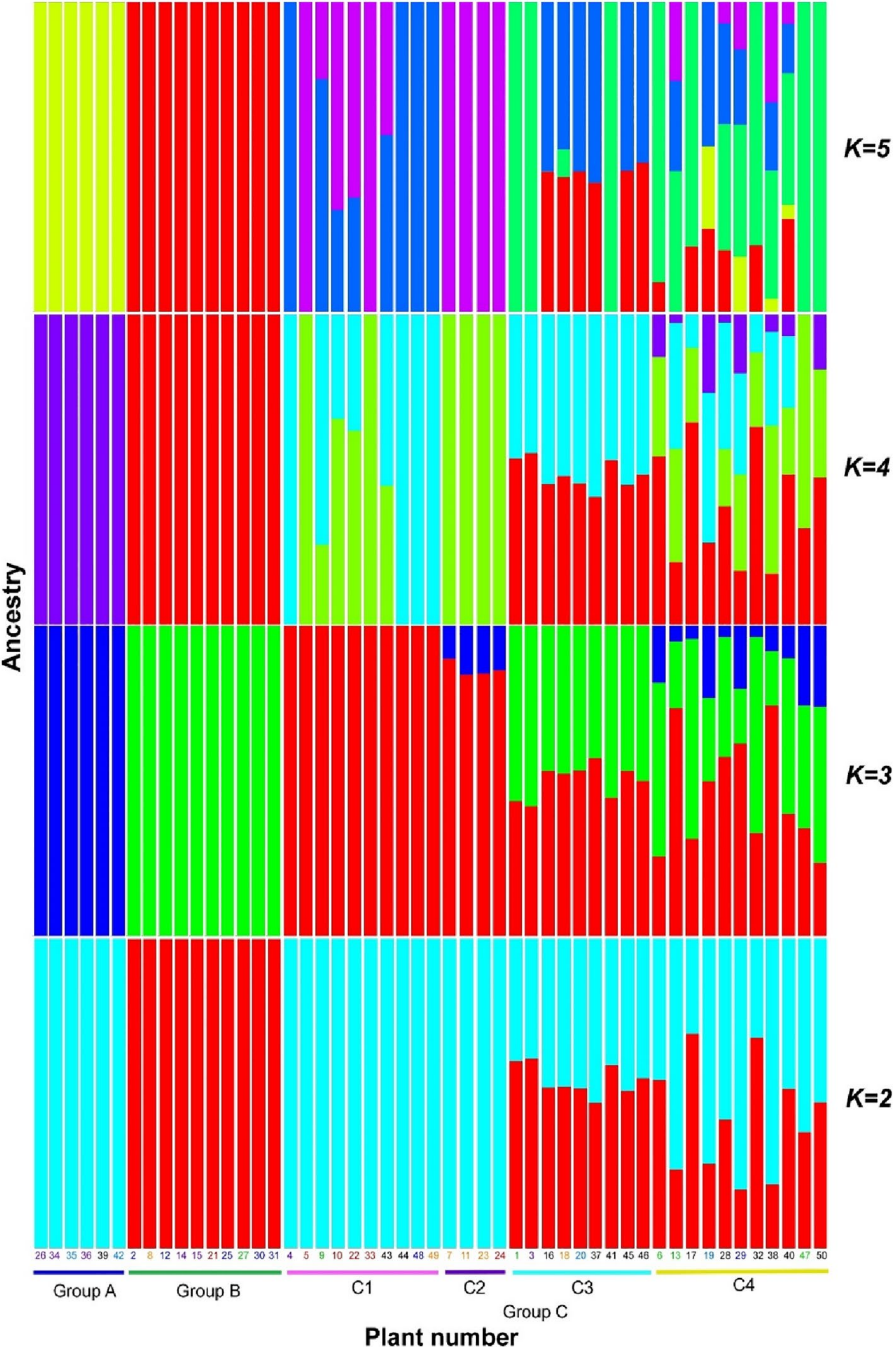
Admixture analysis of *Allium macrostemon* samples used in this study, illustrated by *K* = 2–5 admixture plots. The horizontal axis displays both the group names and sample numbers. The colour schemes of the samples and the groupings correspond to those in Figs. 1 and 2, respectively. It should be noted that the color used for genetic clusters differs from that used for group names and sample numbers. This figure was generated using R software (version 4.1.2).

We performed an admixture analysis to estimate the degree of genetic mixing. We used values from 1 to 10 as possible *K* values (symbolizing the number of ancestral populations), computed the cross-validation (CV) errors, and estimated the most likely *K* value, i.e., the *K* value with the lowest CV (Supplementary Fig. S2). The CV error was minimized at *K* = 3, indicating that the most likely number of ancestral populations is three, aligning with the number of groups identified above. The results of the admixture analysis are displayed (Fig. 5, Supplementary Fig. S3). The admixture analysis detected no gene flow into groups A and B from other groups. Conversely, this analysis identified gene flow into group C from either group A, group B, or both.

### Group C consists of four subgroups

Analysis of the results at *K* = 3, the highest possible value, divided the individuals into four subgroups: subgroup C1, with no detected gene flow from either group A or group B (plants 4, 5, 9, 10, 22, 33, 43, 44, 48, and 49); subgroup C2, with detected gene flow only from group A (plants 7, 11, 23, and 24); subgroup C3, with detected gene flow only from group B (plants 1, 3, 16, 18, 20, 37, 41, 45, and 46); and subgroup C4, with detected gene flow from both groups A and B (plants 6, 13, 17 (and its technical replicate 32), 19, 28, 29, 38, 40, 47, and 50). Subgroups C1, C2, and C3 were also found in the PCA and MDS analyses. Cluster analysis demonstrated that subgroups C1, C2, and C3 formed one or two clusters. Additionally, subgroup C1 was the most distant from groups A and B in the PCA and MDS analyses. Within each subgroup, we did not identify characteristics specific to particular regions. The admixture analysis also displayed more intense gene flow into group C from group B than from group A.

### Statistical interpretations of the classifications

We performed a statistical analysis on the six populations. The *Fst* value analysis (Table 2) exposed a high level of genetic differentiation between groups A and B, yet a comparatively low average degree of genetic differentiation between group A and subgroups C1, C2, C3, or C4, as well as between group B and any of these subgroups. Notably, the genetic differentiation between group B and either C1 or C2 was higher than that between group A and either C1 or C2. Conversely, the *Fst* values between group B and either C3 or C4 were lower compared to those between group A and either C3 or C4.

**Table 2:** *Fst* values among the six groups.

	Group B	Group C1	Group C2	Group C3	Group C4
Group A	0.300503	0.159082	0.176953	0.163133	0.124971
Group B		0.170023	0.211171	0.070683	0.076914
Subgroup C1			0.059202	0.062329	0.053722
Subgroup C2				0.089261	0.053697
Subgroup C3					0.021505

For each population, we conducted a statistical analysis. The average values of nucleotide diversity (π) and mean expected heterozygosity (*He*) were higher in the subgroups of group C; these values in groups A and B were similar and lower than in group C (Table 3, Supplementary Table S3). A negative *Fis* value indicates avoidance of inbreeding in groups A, B, and C2, while the relatively high *Fis* values in groups C1, C3, and C4 imply a moderate degree of inbreeding (Table 4).

**Table 3:** Population genetic statistics of each group.

	Mean expected heterozygosity (*He*)	Mean value of π	Mean measure of *Fis*
Group A	0.14653	0.16737	− 0.10965
Group B	0.15041	0.16267	− 0.14291
Group C1	0.20474	0.22111	0.04932
Group C2	0.15921	0.19686	− 0.04108
Group C3	0.21477	0.23195	0.0379
Group C4	0.22037	0.23575	0.12327

**Table 4:** Conservation of heterozygous loci positions across pairs of samples in each group. Some pairs were selected and shown. Full data are presented in Supplementary Table S5.

Group	Plant	Plant	Number of variable sites	Number of conserved heterozygous sites	%
A	No.26 (Shikoku)	No.39 (Kyushu)	12,344	5574	45.1
	No.34 (Shikoku)	No.36 (Shikoku)	15,116	6166	40.8
	No.35 (Chugoku)	No.39 (Kyushu)	11,135	4961	44.5
	No.35 (Chugoku)	No.42 (Chugoku)	17,896	6545	36.6
	No.39 (Kyushu)	No.34 (Shikoku)	9726	5065	52.1
	No.39 (Kyushu)	No.35 (Chugoku)	11,015	5078	46.1
	No.42 (Chugoku)	No.34 (Shikoku)	14,962	5987	40.0
B	No.2 (Kanto)	No.8 (Chubu)	6160	2810	45.6
	No.2 (Kanto)	No.21 (Tohoku)	9316	3695	39.6
	No.8 (Chubu)	No.12 (Kanto)	10,640	4336	40.7
	No.8 (Chubu)	No.27 (Kinki)	7444	3702	49.7
	No.12 (Kanto)	No.14 (Shikoku)	22,672	10,740	47.4
	No.14 (Shikoku)	No.15 (Shikoku)	16,321	7997	49.0
	No.30 (Kanto)	No.31 (Kanto)	14,445	6882	47.6
C1	No.4 (Shikoku)	No.5 (Tohoku)	11,062	1644	14.9
	No.10 (Tohoku)	No.22 (Tohoku)	10,348	4981	48.1
	No.22 (Tohoku)	No.44 (Kyushu)	24,017	4014	16.7
	No.33 (Tohoku)	No.43 (Kyushu)	34,734	5058	14.6
	No.43 (Kyushu)	No.4 (Shikoku)	19,758	2748	13.9
	No.48 (Kanto)	No.4 (Shikoku)	7961	4030	50.7
	No.49 (Chubu)	No.4 (Shikoku)	11,033	2402	21.8
C2	No.7 (Chubu)	No.11 (Chubu)	13,991	3218	23.0
	No.7 (Chubu)	No.23 (Chubu)	15,078	3307	21.9
	No.11 (Chubu)	No.23 (Chubu)	15,539	4533	29.2
	No.11 (Chubu)	No.24 (Tohoku)	13,055	7026	53.8
	No.23 (Chubu)	No.24 (Tohoku)	16,974	4967	29.3
	No.24 (Tohoku)	No.7 (Chubu)	15,418	3432	22.2
C3	No.1 (Kinki)	No.3 (Shikoku)	13,815	5548	40.1
	No.3 (Shikoku)	No.20 (Chugoku)	20,615	3629	17.6
	No.16 (Kyushu)	No.20 (Chugoku)	25,155	5146	20.4
	No.18 (Chubu)	No.41 (Kyushu)	12,343	2167	17.5
	No.37 (Kyushu)	No.45 (Kyushu)	11,183	2933	26.2
	No.45 (Kyushu)	No.1 (Kinki)	13,057	2782	21.3
	No.46 (Kyushu)	No.16 (Kyushu)	25,504	6161	24.1
C4	No.6 (Kinki)	No.13 (Kinki)	24,481	3843	15.7
	No.13 (Kinki)	No.28 (Kyushu)	26,763	3199	11.9
	No.17 (Okinawa)	No.32 (Okinawa)	12,418	5361	43.9
	No.19 (Chugoku)	No.38 (Kyushu)	20,175	2568	12.7
	No.29 (Shikoku)	No.47 (Kinki)	3007	131	4.3
	No.47 (Kinki)	No.6 (Kinki)	2473	136	5.4
	No.50 (Kyushu)	No.6 (Kinki)	12,063	2425	20.1

We calculated the ratio of heterozygous loci for each individual (Supplementary Table S4). Ten individuals exhibited a relatively low ratio of heterozygous loci, with a value below 0.2. All ten plants (5, 7, 17, 28, 29, 33, 38, 43, 47, and 50) belonged to group C.

### Both groups A and B propagated asexually, while group C propagated sexually

*A. macrostemon* reproduces asexually via bulbs, particularly with human intervention. We detected asexual reproduction in the plant by examining the conservation of heterozygous loci positions via pairwise alignments. The degrees of conservation for the technical replicates, plants 14 and 15, 30 and 31, and 17 and 32, were 49.0%, 47.6%, and 43.9%, respectively. De novo analysis of RAD-Seq data, which contains many missing values, is likely to result in 40–50% conservation between somatic clones. Consequently, we determined the presence or absence of asexual reproduction by setting the value of conservation at nearly 40% as the criterion. It should be noted that a similar value of conservation was also used as a criterion for clonal propagation in a study of Japanese pepper that used de novo analysis of RAD-Seq data^28^.

We investigated the conservation levels among plants in each group, both within and across districts (Table 4, Supplementary Table S5). The conservation level in group A was high, with over half of the pairs showing above 40% (40.8–52.1%). Some pairs had conservation levels slightly below 40% (36.6–39.7%). However, given the somatic mutations (including loss of heterozygosity) that occur during asexual reproduction, it is likely that these plants with slightly lower conservation values also propagated from the same ancestor via asexual reproduction. The conservation level in group B was high, with all pairs of plants showing an average of more than 40% (40.7–49.7%) except for one pair which showed 39.6%. Conversely, nearly all the pairs observed in group C showed a low level of conservation, with only six exceptions. Thus, groups A and B propagated from a single plant by asexual reproduction, whereas the members of group C propagated almost exclusively without asexual reproduction.

### Morphological differences exist among ancestral populations A, B, and C1

**Figure 6:**
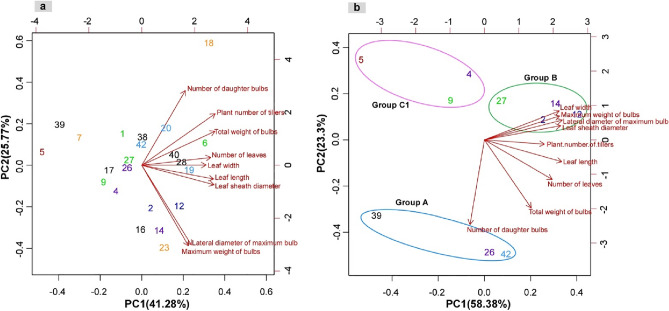
Principal component analysis (PCA) of morphological data from samples of *Allium macrostemon.* (**a**) analysis of all 22 samples, (**b**) analysis of 10 samples belonging to groups A, B, and C1. The contribution rate of each principal component is indicated in parentheses. The colour schemes of the samples and the groupings correspond to those in Figs. 1 and 2, respectively. The figure was generated using R software (version 4.1.3).

Out of the 47 individuals subjected to RAD-Seq, 22 were cultured and analyzed for morphological data using nine distinct measurements (Table 5). Subsequently, we conducted a principal component analysis (PCA) on the 22 cultured individuals, using the acquired morphological data (Fig. 6a). Given that groups A, B, and C1 represent ancestral populations, a separate PCA was performed exclusively on the ten individuals from these groups (Fig. 6b). The first PCA that included all 22 individuals accounted for 41.28% of the total variance, with the second principal component contributing 25.8%. Conversely, in the PCA exclusive to the ten selected individuals, the first and second principal components accounted for 58.4% and 23.3% of the variance, respectively. Notably, while the graphical representation in Fig. 6a did not clearly delineate genetic or regional grouping, Fig. 6b distinctly showcased genetic groupings. These observations underscore the presence of morphological disparities among the ancestral populations A, B, and C1.

**Table 5:** Morphological features of *Allium macrostemon* collected in various parts of Japan.

No	Isolate	Group	Number of leaves	Plant number of tillers	Leaf length	Leaf width	Leaf sheath diameter	Number of daughter bulbs	Total weight of bulbs	Maximum weight of bulbs	Lateral diameter of maximum bulb
39	WOF366	A	7.3 ± 1.3	2.5 ± 1.3	44.8 ± 0.2	5.1 ± 0.4	8.5 ± 0.0	16.7 ± 3.3	19.8 ± 4.0	3.9 ± 0.0	19.7 ± 0.2
26	WOKW581	A	10.4 ± 0.8	2.0 ± 0.2	58.0 ± 4.3	6.9 ± 0.6	10.5 ± 0.5	15.6 ± 0.0	30.2 ± 0.6	7.7 ± 0.1	23.8 ± 0.0
42	WOOY575	A	9.3 ± 0.1	3.4 ± 1.6	58.7 ± 0.2	7.8 ± 0.6	12.5 ± 0.6	16.9 ± 3.5	31.5 ± 1.7	6.4 ± 2.1	22.1 ± 2.0
2	WOGU466	B	8.4 ± 0.0	4.0 ± 0.4	61.4 ± 0.9	7.9 ± 0.3	12.9 ± 0.1	6.0 ± 0.4	22.1 ± 0.9	11.4 ± 0.9	26.8 ± 0.4
12	WOIB449	B	9.9 ± 0.9	4.1 ± 0.7	58.9 ± 3.4	9.6 ± 1.5	16.2 ± 2.0	5.6 ± 0.4	23.6 ± 3.4	10.6 ± 0.4	26.9 ± 0.2
14	WOKO594	B	9.1 ± 0.7	3.0 ± 0.0	56.9 ± 1.9	8.2 ± 0.6	14.4 ± 0.1	5.0 ± 0.0	24.3 ± 2.3	13.3 ± 3.6	28.6 ± 2.3
27	WOME413	B	9.4 ± 1.2	4.3 ± 1.3	56.0 ± 5.0	8.5 ± 1.3	11.3 ± 1.5	4.6 ± 2.8	12.2 ± 5.6	6.9 ± 1.3	22.6 ± 1.8
5	WOA360	C1	5.1 ± 0.3	1.5 ± 0.1	45.7 ± 3.1	6.2 ± 0.0	7.4 ± 0.2	3.4 ± 0.2	8.5 ± 0.2	4.9 ± 0.1	19.5 ± 0.7
4	WOEH562	C1	7.6 ± 1.2	1.9 ± 0.5	49.3 ± 3.5	8.0 ± 0.5	14.6 ± 2.3	4.7 ± 1.1	15.9 ± 0.3	7.9 ± 1.7	23.9 ± 1.2
9	WONR546	C1	8.5 ± 1.3	1.8 ± 0.6	49.4 ± 0.7	7.6 ± 0.6	10.3 ± 2.2	4.6 ± 0.8	17.1 ± 0.4	7.3 ± 1.0	24.2 ± 0.5
23	WOIS532	C2	9.4 ± 0.2	2.5 ± 0.3	62.3 ± 1.9	6.3 ± 0.4	12.0 ± 0.5	9.2 ± 1.4	44.6 ± 22.6	16.4 ± 8.6	31.4 ± 5.7
7	WOTY540	C2	9.4 ± 2.1	2.0 ± 0.2	38.5 ± 3.8	5.3 ± 0.5	9.9 ± 0.6	14.0 ± 1.8	27.2 ± 8.0	4.8 ± 0.7	21.3 ± 0.1
18	WOFI554	C3	9.2 ± 0.4	8.5 ± 0.7	54.5 ± 0.8	8.7 ± 0.4	13.9 ± 0.1	76.3 ± 17.7	70.3 ± 0.7	5.6 ± 1.1	21.5 ± 1.4
16	WOKG195	C3	7.5 ± 0.7	3.5 ± 0.1	45.3 ± 2.2	7.1 ± 0.2	13.6 ± 0.8	6.1 ± 0.7	33.6 ± 1.8	14.3 ± 0.6	29.6 ± 0.4
1	WOKY536	C3	8.0 ± 1.2	5.8 ± 0.0	52.2 ± 3.9	8.1 ± 0.2	10.0 ± 1.7	10.9 ± 0.7	24.4 ± 1.8	5.8 ± 0.2	22.0 ± 0.4
20	WOOY574	C3	12.0 ± 2.4	5.5 ± 0.9	51.8 ± 4.5	7.9 ± 0.1	15.6 ± 1.5	13.2 ± 0.2	30.1 ± 14.9	5.4 ± 1.9	20.9 ± 1.9
40	WOF395	C4	9.1 ± 2.5	5.4 ± 2.8	53.7 ± 3.4	8.5 ± 0.3	14.9 ± 1.6	18.7 ± 11.3	45.9 ± 1.9	8.4 ± 2.4	24.9 ± 1.8
38	WOM366	C4	8.2 ± 0.6	3.3 ± 0.7	51.0 ± 0.9	7.5 ± 0.4	13.3 ± 1.9	20.8 ± 0.2	50.3 ± 17.1	7.3 ± 2.5	22.8 ± 2.5
28	WOO199	C4	9.7 ± 2.3	6.1 ± 0.7	69.6 ± 6.4	6.8 ± 0.8	13.9 ± 1.5	13.6 ± 0.8	44.7 ± 4.7	8.7 ± 1.0	25.2 ± 0.5
17	WOOK122	C4	7.2 ± 0.6	2.3 ± 0.3	53.5 ± 2.7	6.3 ± 0.2	11.0 ± 0.4	14.7 ± 0.3	32.6 ± 0.7	7.1 ± 0.7	25.0 ± 2.2
6	WOSG538	C4	10.7 ± 1.5	6.2 ± 0.2	76.0 ± 8.7	8.1 ± 0.8	10.6 ± 1.8	27.0 ± 8.0	57.6 ± 0.8	9.7 ± 2.2	25.0 ± 1.0
19	WOT105	C4	11.2 ± 1.2	4.4 ± 0.2	61.5 ± 1.1	7.4 ± 0.2	13.9 ± 0.4	16.7 ± 1.1	61.3 ± 1.2	9.4 ± 0.1	27.0 ± 1.5

Based on the results of the PCA, we further investigated which morphological measurements primarily influenced the groupings observed in Fig. 6b. Among the variables, leaf width, maximum weight of bulbs, lateral diameter of the maximum bulb, leaf sheath diameter, and the number of plant tillers were characterized by elevated values in group B. Conversely, the number of daughter bulbs demonstrated higher values within group A. Leaf length, the number of leaves, and the total weight of bulbs displayed enhanced values in both group A and group B, indicating shared morphological features among these two groups. Remarkably, none of the measurements presented high values for group C1, suggesting distinct morphological characteristics within this group.

We examined the preceding study^33^ that analyzed the concentration of phenolics in *A. macrostemon* collected from various regions across Japan. Using data extracted from this study^33^, we conducted a PCA, employing the quantitative values of functional components as variables. However, similar to the results in Fig. 6a, this PCA did not facilitate the delineation of samples based on genetic or regional clusters.

## Discussion

The key revelation of this study is the historical relevance of *A. macrostemon*, a plant currently overlooked in Japan but widely used in earlier times. With minimal exceptions, no distinct regional clusters were discernible across all groups. Previous reports have noted the lack of a clear correlation between the plant's collection sites and the content of flavonoids and ferulic acid glycosides^18, 19, 33^. This absence of phenotypic differentiation between various collection sites aligns with the genetic findings of our study. These results suggest that although the plant's inter-regional migration is currently inactive, it was once actively transported, with its bulbous nature potentially facilitating this movement. Its historical significance is underscored by its wide usage as both a vegetable and medicinal resource.

The previously active utilization of this plant influence our interpretation of classical Japanese literature. Mentions of the plant are found in Japanese literary works such as the Kojiki and Manyoshu. Modern interpretations often suggest that individuals in the past had a fondness for wild plants. However, given the active use of *A. macrostemon* as a food source, it is plausible that emperors and poets from these historical periods may have appreciated it not merely as a wild plant but as a cultivated vegetable. This potential reevaluation offers a more nuanced understanding of cultural and lifestyle patterns in historic Japan.

Furthermore, this study revealed that *A. macrostemon* exhibits two distinct reproductive strategies. Groups A and B, which were clearly established as discrete groups, demonstrated evidence of clonal propagation based on the analysis of the conservation of heterozygous loci positions. This suggests that each group represents a population formed primarily through asexual reproduction. The negative *Fis* values observed in groups A and B are also likely related to this clonal propagation (Table 3). Therefore, the principal mode of reproduction within groups A and B appears to be through bulb propagation, highlighting a notable asexual reproduction strategy.

Conversely, group C exhibited a more complex population structure and demonstrated a largely non-clonal mode of propagation, with a few exceptions (Table 4). This suggests that group C primarily employs an alternative, bulb-independent mode of reproduction, most likely through hybridization leading to seed formation. Within group C, several members demonstrated notably low heterozygosity. This shift towards homozygosity is likely related to the reproductive dynamics brought about by hybridization.

Our study subdivided group C into four subgroups. Among them, subgroup C1 showed no signs of gene flow from either group A or group B. Furthermore, there was no evidence of clonal propagation within subgroup C1. Thus, group A, group B, and subgroup C1 represent distinct ancestral populations. Gene flow from group A, group B, or both, was observed in other subgroups within group C, contributing to increased genetic diversity within this group (Fig. 5). The possibility of gene flow due to human intervention cannot be discounted, but natural hybridization seems more probable. Human activities, including the movement of plants and bulbs, might have heightened the chances of natural hybridization between groups. This implies that human-induced translocation of this plant might have facilitated the augmentation of its genetic diversity. Consequently, this observation suggests that incidental human intervention may escalate the genetic diversity of a plant species, even one that can also propagate in the wild.

We conducted a cultivation trial to ascertain the morphological traits of this plant's genetic resources in Japan. Although we observed morphological variations, a discernible pattern remained elusive, prompting us to withhold the publication of these results. Nevertheless, the RAD-Seq analysis uncovered that groups A, B, and C1 formed the ancestral populations. Consequently, we re-evaluated only these three groups based on morphological characteristics, resulting in their partition into three distinct clusters. Essentially, excluding the population resulting from hybridization, Japanese *A. macrostemon* was genetically and morphologically divided into three groups. It was not possible to classify the three ancestral groups based on differences in their functional components. Still, we remain open to the possibility that future research may uncover componential differences among three groups. If ongoing research finds no compositional characteristics distinguishing the three ancestral groups, and if historical records indicate distinct recognition of each group, it might suggest that past populations valued this plant more for its vegetable properties than its medicinal benefits.

Given its widespread presence in Japan, *A. macrostemon* might have originated locally or been introduced from overseas, either in its entirety or in parts. Notably, groups A and B, which primarily reproduce through bulb propagation—a method well-suited for human-assisted migration, might have been introduced from foreign regions. In this scenario, the most desirable varieties might have been selected overseas and then imported into Japan. Conversely, group C, which displays a bulb-independent mode of reproduction, could be endemic to Japan. This group's unique reproductive strategy may have evolved to adapt to the specific environmental conditions prevalent in Japan, suggesting a native origin. However, more comprehensive research is required to conclusively determine the plant's origin and the pathways of its spread.

Interestingly, the Japanese word 'no-biru' also has another name, 'chosen no-biru'. 'Chosen' means Korea, suggesting that part of this plant may have been introduced to Japan from Korea^34^. However, 'chosen' also has other meanings. 'Chosen' is used as a prefix for closely related organisms, like different species within the same genus. It is therefore possible that the plant was previously differentiated into two species. Although it remains unclear whether 'chosen' refers to Korea or to a closely related organism, the possibility that people in the past distinguished at least two species of this plant is intriguing and suggests a link with the grouping in this study. The existence of morphological differences among ancestral populations A, B, and C1 also supports this possibility. Further studies analyzing individuals from China and Korea, which would allow us to speculate on the evolution of this plant and the route of its arrival in Japan, would provide further clarification.

A critical reference for discussing the potential foreign introduction of *A. macrostemon* is our prior study on the scallion mosaic virus (ScaMV) in the family *Potyviridae* that infects this plant species^35^. This earlier research revealed that the genome types of ScaMV found in Japan are dispersed among various genomic patterns, suggesting multiple introductions into Japan via different routes. Furthermore, it was shown that the parent ScaMV genomes recombined relatively recently in Japan and were subsequently spread across various districts by human activities. This supports the speculation that the plant might have been established in Japan prior to the introduction of the virus. Nevertheless, it does not entirely exclude the possibility that the plant and the virus were introduced simultaneously as part of the same event. In essence, this underlines the complex historical and ecological interplays between *A. macrostemon*, its associated viruses, and human activities.

In conclusion, our findings provide valuable insights into the genetic resources of *A. macrostemon* collected across Japan. Through genetic-level investigations, we have provided evidence suggesting past human-induced migration of this species. In this study, the use of RAD-Seq technology was instrumental in analyzing the genetic diversity of a species without prior genomic information, similar to what has been done previously with Japanese pepper^28^. Looking forward, conservation efforts for this plant's genetic resources will require attention to not only genetic diversity but also key traits such as the chemical compounds present in the plant's bulbs and leaves, which possess potential value for culinary uses.

## Methods

### Plant materials

A total of 47 wild Japanese garlic (*A. macrostemon*) samples were collected from various sites along riverbanks and fields across Japan. We searched for wild Japanese garlic plants on foot and by car. All methods involving plants were carried out in accordance with relevant guidelines and regulations. Plants were collected with permission from their owners. All wild Japanese garlics were maintained at the Center for Education and Research in Agricultural Innovation, Saga University (Saga, Japan). Detailed information about each plant sample, its origin, and the respective collection date is provided in Table 1. Certain samples served as technical replicates of the same plant, samples 30 and 31, samples 14 and 15, as well as samples 17 and 32.

### Cultivation trial and measurement

The cultivation trial of *A. macrostemon* was conducted in a field (grey lowland soil, pH 6.1, EC 0.19 mS cm^−1^) located at Saga University (latitude 33° 30′ 91.308″ N; longitude 130° 33′ 28.119″ W). The compound of fertilizer (N:P_2_O_5_:K_2_O = 14:14:14%) was applied at 8 kg of N, phosphate, and potassium each at 10 a^−1^, and the field was covered with black mulch. The bulbs were planted on 9 October 2015 and 12 October 2016. The planting density was set with a 150 cm row width and 25 cm spacing between plants both lengthwise and widthwise. Five medium-scale plants of the isolate were arranged per row, with one plant per hole. We recorded various morphological parameters over a 2-year period (2016 and 2017), including the number of leaves, plant number of tillers, leaf length and width, leaf sheath diameter, the number of daughter bulbs, the total and maximum weight of bulbs, and the lateral diameter of the maximum bulb.

### DNA extraction and double-digest restriction site amplified DNA sequencing (ddRAD-Seq)

The extraction and purification of DNA were performed using the CTAB method^36^, followed by RNase treatment. The quality of the isolated genomic DNA was validated through 0.8% agarose gel electrophoresis. The DNA concentration was quantified using a Qubit dsDNA BR Assay Kit (Invitrogen, MA, USA). The library for ddRAD-Seq was prepared based on the method of Sakaguchi et al.^37^, with some adaptations from the original protocol^25^. The libraries were sequenced using 51-bp single-end reads at Macrogen (Seoul, Korea) on one lane of a HiSeq 2000 (Illumina, San Diego, CA, USA).

### De novo mapping and variant calling

The denovo_map.pl script from the Stacks package (version 2.60)^38^, a wrapper for ustacks, sstacks, tsv2bam, and gstacks, was used to map reads de novo without using a reference genome sequence. The options for denovo_map.pl were -M 4 (number of mismatches allowed between stacks within individuals [for ustacks]), -n 4 (number of mismatches allowed between stacks between individual [for cstacks]), and -m 3 (number of identical reads required to initiate a new putative allele [for ustacks]). Genotyping data were created by assigning one individual per population to specify all the samples. After performing denovo_map.pl, the populations program of the Stacks package (version 2.60) created the vcf (variant call format)^39^, plink^40^, and phylip files using the -R 0.5 (minimum percentage of individuals across populations required to process a locus), -write-single-snp (restrict data analysis to only the first SNP per locus), -min-maf 0.05 (minimum minor allele frequency required to process a nucleotide site at a locus), -vcf, -plink, and -phylip-var-all options.

### Principal component analysis, multidimensional scaling analysis, and cluster analysis

PCA and MDS analyses were performed using the vcf file generated by the populations program. The SNPRelate program^41^ in the R software environment (version 4.1.2) was employed to convert the vcf file into a gds (genomic data structure) file and generate PCA and MDS plots. The contribution ratio for each principal component was also computed. Only bi-allelic loci were included in this analysis. The SNPRelate package was further used to construct a dendrogram, calculating identity by state (IBS) pairwise distances. Image outputs were produced using the basic functions within the R software environment.

### Principal component analysis of morphological test and functional component analysis

PCA of morphological tests and functional component analysis were performed using R software (version 4.1.3). Input data for these analyses were sourced from CSV files containing tables of morphological tests or functional component results. The R software (version 4.1.3) was used to calculate important component and generate PCA plots. The contribution ratio for each principal component was also computed. The diagram outputs were produced using the basic functions within the R software environment.

### Admixture analysis

The plink program (plink 2, version 1.90p)^40^ was used to generate the input files required for the admixture program. The admixture program (version 1.3.0)^42^ was utilized to determine the admixture history and cross-validation (CV) error for the hypothetical runs from *K* (number of ancestral populations) = 1–10. The CV error plot was drawn using the received log data to ascertain the optimal *K* value. The R software (version 4.1.2) was utilized to create admixture plots using the Q estimate files generated by the admixture program.

### Statistical analysis

Groups differentiated by the afore mentioned analyses were designated as separate populations in the population map data required for the Stacks package (version 2.60)^38^. The denovo_map.pl script was re-executed following data modifications in the population map. The parameters used to run the populations program included -R 0.7, -min-maf 0.05, -write-single-snp, -fstats, and -fst-correction.

### Analysis of asexual reproduction

The occurrence of asexual reproduction was verified by examining the conservation of heterozygous loci positions using pairwise alignments. The populations program was also used to create pairwise alignment between the two individuals. In this study, the -R option was set to one. The percentage of conserved heterozygous loci was calculated by dividing the number of conserved sites by the total number of variant sites (provided by the populations program).

### English writing

The chatGPT was used solely for the purpose of improving English writing.

### Supplementary Information

Supplementary Information.

## Data Availability

Sequences are available at the DNA Data Bank of Japan Sequence Read Archive (https://ddbj.nig.ac.jp/resource/sra-submission/DRA013735; Accession no. DRA013735).
