# Supplementary material for: RAD-Seq analysis of wild Japanese garlic (Allium macrostemon Bunge) growing in Japan revealed that this neglected crop was previously actively utilized
Source: Sci Rep. 2023 Sep 29;13:16354. doi: 10.1038/s41598-023-43537-5 (PMC10541398; doi:10.1038/s41598-023-43537-5)
Supplement: Supplementary file 1 — Supplementary Information. [file 41598_2023_43537_MOESM1_ESM.doc]

**RAD-Seq analysis of wild Japanese garlic (*Allium macrostemon* Bunge) growing in Japan revealed that this neglected crop was previously actively utilized**

Wiwit Probowati^1,2,3^, Shogo Koga^2,3^, Kentaro Harada^2,3^, Yukio Nagano^1,4^, Atsushi J. Nagano^5,6^, Kanji Ishimaru^1,3^, Kazusato Ohshima^1,3^, Shinji Fukuda^1,2,3✉^

^1^ The United Graduate School of Agricultural Sciences, Kagoshima University, 1-21-24 Korimoto, Kagoshima 890-0065, Japan

^2^ Center for Education and Research in Agricultural Innovation, Saga University, Saga, Japan.

^3^ Faculty of Agriculture, Saga University, Saga, Japan.

^4^ Analytical Research Center for Experimental Sciences, Saga University, Saga, Japan.

^5^ Faculty of Agriculture, Ryukoku University, Otsu, Japan

^6^ Institute for Advanced Biosciences, Keio University, Tsuruoka, Yamagata, Japan.

^✉^email: [sfukuda@cc.saga-u.ac.jp](mailto:sfukuda@cc.saga-u.ac.jp)

| **Supplementary Table S1.** Number of filtered reads for each sample. | | |
| --- | --- | --- |
| Sample No. | Number of filtered reads |  |
| 1 | 761,434 |  |
| 2 | 222,798 |  |
| 3 | 668,064 |  |
| 4 | 430,536 |  |
| 5 | 241,687 |  |
| 6 | 736,658 |  |
| 7 | 447,758 |  |
| 8 | 259,664 |  |
| 9 | 606,686 |  |
| 10 | 388,167 |  |
| 11 | 559,505 |  |
| 12 | 2,482,219 |  |
| 13 | 2,077,219 |  |
| 14 | 1,129,355 |  |
| 15 | 732,237 |  |
| 16 | 1,075,039 |  |
| 17 | 486,450 |  |
| 18 | 1,464,978 |  |
| 19 | 584,580 |  |
| 20 | 1,401,298 |  |
| 21 | 1,088,044 |  |
| 22 | 562,117 |  |
| 23 | 671,865 |  |
| 24 | 694,943 |  |
| 25 | 919,017 |  |
| 26 | 1,619,164 |  |
| 27 | 372,234 |  |
| 28 | 806,780 |  |
| 29 | 637,460 |  |
| 30 | 894,718 |  |
| 31 | 633,044 |  |
| 32 | 747,819 |  |
| 33 | 2,580,109 |  |
| 34 | 520,879 |  |
| 35 | 855,633 |  |
| 36 | 1,962,933 |  |
| 37 | 474,560 |  |
| 38 | 772,967 |  |
| 39 | 379,968 |  |
| 40 | 1,785,853 |  |
| 41 | 227,737 |  |
| 42 | 1,513,493 |  |
| 43 | 2,010,432 |  |
| 44 | 3,323,906 |  |
| 45 | 333,527 |  |
| 46 | 1,901,266 |  |
| 47 | 175,864 |  |
| 48 | 291,968 |  |
| 49 | 298,701 |  |
| 50 | 304,332 |  |
| Total | 46,117,665 |  |
| Average reads | 922,353 |  |

| **Supplementary Table S2.** Depth of coverage for processed samples. | | |
| --- | --- | --- |
| Sample No. | Depth of coverage |  |
| 1 | 16.63x |  |
| 2 | 10.02x |  |
| 3 | 14.49x |  |
| 4 | 12.26x |  |
| 5 | 10.70x |  |
| 6 | 15.03x |  |
| 7 | 12.67x |  |
| 8 | 10.36x |  |
| 9 | 13.62x |  |
| 10 | 11.52x |  |
| 11 | 12.33x |  |
| 12 | 27.48x |  |
| 13 | 26.31x |  |
| 14 | 16.98x |  |
| 15 | 15.06x |  |
| 16 | 16.33x |  |
| 17 | 12.92x |  |
| 18 | 21.64x |  |
| 19 | 13.76x |  |
| 20 | 22.31x |  |
| 21 | 18.49x |  |
| 22 | 13.16x |  |
| 23 | 14.21x |  |
| 24 | 14.01x |  |
| 25 | 17.00x |  |
| 26 | 23.61x |  |
| 27 | 11.63x |  |
| 28 | 16.32x |  |
| 29 | 14.03x |  |
| 30 | 15.56x |  |
| 31 | 13.92x |  |
| 32 | 15.79x |  |
| 33 | 32.30x |  |
| 34 | 12.77x |  |
| 35 | 16.65x |  |
| 36 | 26.46x |  |
| 37 | 12.45x |  |
| 38 | 14.84x |  |
| 39 | 11.85x |  |
| 40 | 25.10x |  |
| 41 | 10.23x |  |
| 42 | 22.78x |  |
| 43 | 24.56x |  |
| 44 | 27.93x |  |
| 45 | 11.08x |  |
| 46 | 25.40x |  |
| 47 | 30.93x |  |
| 48 | 11.25x |  |
| 49 | 11.06x |  |
| 50 | 11.38x |  |
| Average reads | 16.78x |  |

**Supplementary Table S3.** Population genetic statistics (Content of the "populations.sumstats_summary.tsv" that was created by Stacks program).

| # Variant positions |  |  |  |  |  |  |
| --- | --- | --- | --- | --- | --- | --- |
| # Pop ID | Group A | Group B | Group C1 | Group C2 | Group C3 | Group C4 |
| Private | 181 | 26 | 60 | 7 | 14 | 45 |
| Num_Indv | 5.1322 | 8.4892 | 7.51666 | 3.22413 | 7.17555 | 8.01691 |
| Var | 1.47102 | 2.96184 | 2.99344 | 0.84313 | 2.02383 | 2.11466 |
| StdErr | 0.01607 | 0.02253 | 0.02261 | 0.01209 | 0.01859 | 0.019 |
| P | 0.8748 | 0.87244 | 0.85281 | 0.87242 | 0.84468 | 0.84597 |
| Var | 0.03627 | 0.03609 | 0.02316 | 0.0317 | 0.02381 | 0.02012 |
| StdErr | 0.00252 | 0.00249 | 0.00199 | 0.00234 | 0.00202 | 0.00185 |
| Obs_Het | 0.22758 | 0.23905 | 0.20372 | 0.22118 | 0.22235 | 0.19436 |
| Var | 0.13206 | 0.13606 | 0.05854 | 0.11139 | 0.06394 | 0.04576 |
| StdErr | 0.00482 | 0.00483 | 0.00316 | 0.00439 | 0.0033 | 0.0028 |
| Obs_Hom | 0.77242 | 0.76095 | 0.79628 | 0.77882 | 0.77765 | 0.80564 |
| Var | 0.13206 | 0.13606 | 0.05854 | 0.11139 | 0.06394 | 0.04576 |
| StdErr | 0.00482 | 0.00483 | 0.00316 | 0.00439 | 0.0033 | 0.0028 |
| Exp_Het | 0.14653 | 0.15041 | 0.20474 | 0.15921 | 0.21477 | 0.22037 |
| Var | 0.04479 | 0.04444 | 0.03428 | 0.0419 | 0.03401 | 0.02863 |
| StdErr | 0.0028 | 0.00276 | 0.00242 | 0.00269 | 0.00241 | 0.00221 |
| Exp_Hom | 0.85347 | 0.84959 | 0.79526 | 0.84079 | 0.78523 | 0.77963 |
| Var | 0.04479 | 0.04444 | 0.03428 | 0.0419 | 0.03401 | 0.02863 |
| StdErr | 0.0028 | 0.00276 | 0.00242 | 0.00269 | 0.00241 | 0.00221 |
| Pi | 0.16737 | 0.16267 | 0.22111 | 0.19686 | 0.23195 | 0.23575 |
| Var | 0.05994 | 0.05252 | 0.04027 | 0.06841 | 0.03975 | 0.03288 |
| StdErr | 0.00324 | 0.003 | 0.00262 | 0.00344 | 0.00261 | 0.00237 |
|  |  |  |  |  |  |  |
| # All positions (variant and fixed) | |  |  |  |  |  |
| # Pop ID | Group A | Group B | Group C1 | Group C2 | Group C3 | Group C4 |
| Private | 181 | 26 | 60 | 7 | 14 | 45 |
| Sites | 463307 | 472742 | 473969 | 469990 | 474381 | 474381 |
| Variant_Sites | 5696 | 5836 | 5853 | 5769 | 5856 | 5856 |
| Polymorphic_Sites | 1954 | 2177 | 3738 | 2313 | 3932 | 4458 |
| %Polymorphic_Loci | 0.42175 | 0.4605 | 0.78866 | 0.49214 | 0.82887 | 0.93975 |
| Num_Indv | 5.35198 | 8.78861 | 7.88621 | 3.42323 | 7.65381 | 8.48843 |
| Var | 1.02549 | 2.00226 | 2.98427 | 0.68064 | 1.68947 | 2.07898 |
| StdErr | 0.00149 | 0.00206 | 0.00251 | 0.0012 | 0.00189 | 0.00209 |
| P | 0.99846 | 0.99843 | 0.99818 | 0.99843 | 0.99808 | 0.9981 |
| Var | 0.00064 | 0.00064 | 0.00055 | 0.00059 | 0.00059 | 0.00054 |
| StdErr | 0.00004 | 0.00004 | 0.00003 | 0.00004 | 0.00004 | 0.00003 |
| Obs_Het | 0.0028 | 0.00295 | 0.00252 | 0.00271 | 0.00274 | 0.0024 |
| Var | 0.00225 | 0.00238 | 0.00123 | 0.00196 | 0.00139 | 0.00103 |
| StdErr | 0.00007 | 0.00007 | 0.00005 | 0.00006 | 0.00005 | 0.00005 |
| Obs_Hom | 0.9972 | 0.99705 | 0.99748 | 0.99729 | 0.99726 | 0.9976 |
| Var | 0.00225 | 0.00238 | 0.00123 | 0.00196 | 0.00139 | 0.00103 |
| StdErr | 0.00007 | 0.00007 | 0.00005 | 0.00006 | 0.00005 | 0.00005 |
| Exp_Het | 0.0018 | 0.00186 | 0.00253 | 0.00195 | 0.00265 | 0.00272 |
| Var | 0.00081 | 0.00082 | 0.00093 | 0.00082 | 0.00098 | 0.00095 |
| StdErr | 0.00004 | 0.00004 | 0.00004 | 0.00004 | 0.00005 | 0.00004 |
| Exp_Hom | 0.9982 | 0.99814 | 0.99747 | 0.99805 | 0.99735 | 0.99728 |
| Var | 0.00081 | 0.00082 | 0.00093 | 0.00082 | 0.00098 | 0.00095 |
| StdErr | 0.00004 | 0.00004 | 0.00004 | 0.00004 | 0.00005 | 0.00004 |
| Pi | 0.00206 | 0.00201 | 0.00273 | 0.00242 | 0.00286 | 0.00291 |
| Var | 0.00108 | 0.00097 | 0.00109 | 0.00131 | 0.00115 | 0.00108 |
| StdErr | 0.00005 | 0.00005 | 0.00005 | 0.00005 | 0.00005 | 0.00005 |

**Supplementary Table S4.** Heterozygosity of variant positions of each individual.

| Sample No. | Heterozygosity |
| --- | --- |
| 1 | 0.20357 |
| 2 | 0.20457 |
| 3 | 0.22167 |
| 4 | 0.20953 |
| 5 | 0.17715 |
| 6 | 0.23265 |
| 7 | 0.1904 |
| 8 | 0.21354 |
| 9 | 0.21708 |
| 10 | 0.20147 |
| 11 | 0.22656 |
| 12 | 0.2328 |
| 13 | 0.20741 |
| 14 | 0.24529 |
| 15 | 0.23247 |
| 16 | 0.24403 |
| 17 | 0.1947 |
| 18 | 0.20929 |
| 19 | 0.21214 |
| 20 | 0.20976 |
| 21 | 0.22818 |
| 22 | 0.20296 |
| 23 | 0.2182 |
| 24 | 0.22475 |
| 25 | 0.22925 |
| 26 | 0.2185 |
| 27 | 0.22239 |
| 28 | 0.18518 |
| 29 | 0.17537 |
| 30 | 0.23516 |
| 31 | 0.23666 |
| 32 | 0.2035 |
| 33 | 0.17644 |
| 34 | 0.22353 |
| 35 | 0.21239 |
| 36 | 0.22158 |
| 37 | 0.2284 |
| 38 | 0.17564 |
| 39 | 0.23348 |
| 40 | 0.21307 |
| 41 | 0.22803 |
| 42 | 0.21518 |
| 43 | 0.1879 |
| 44 | 0.21306 |
| 45 | 0.24589 |
| 46 | 0.22616 |
| 47 | 0.02517 |
| 48 | 0.21114 |
| 49 | 0.1984 |
| 50 | 0.16758 |

**Supplementary Table S5.** Conservation of heterozygous loci positions across pairs of samples in each group. Technical replicates are indicated in green.

| Group | Plant | Plant | Number of variable sites | Number of conserved heterozygous sites | % |
| --- | --- | --- | --- | --- | --- |
| A | No.26 | No.36 | 22242 | 8741 | 39.3 |
|  | No.26 | No.39 | 12344 | 5574 | 45.1 |
|  | No.26 | No.42 | 21312 | 8165 | 38.3 |
|  | No.34 | No.35 | 13361 | 5310 | 39.7 |
|  | No.34 | No.36 | 15116 | 6166 | 40.8 |
|  | No.34 | No.39 | 9744 | 5046 | 51.8 |
|  | No.35 | No.26 | 17616 | 6796 | 38.6 |
|  | No.35 | No.39 | 11135 | 4961 | 44.5 |
|  | No.35 | No.42 | 17896 | 6545 | 36.6 |
|  | No.36 | No.35 | 18394 | 6758 | 36.7 |
|  | No.36 | No.39 | 12635 | 5459 | 43.2 |
|  | No.36 | No.42 | 22070 | 8359 | 42.4 |
|  | No.39 | No.34 | 9726 | 5065 | 52.1 |
|  | No.39 | No.35 | 11015 | 5078 | 46.1 |
|  | No.39 | No.42 | 12205 | 5387 | 44.1 |
|  | No.42 | No.34 | 14962 | 5987 | 40.0 |
| B | No.2 | No.8 | 6160 | 2810 | 45.6 |
|  | No.2 | No.14 | 8942 | 4000 | 44.7 |
|  | No.2 | No.21 | 9316 | 3695 | 39.6 |
|  | No.2 | No.25 | 9175 | 3746 | 40.8 |
|  | No.2 | No.27 | 7483 | 3388 | 45.3 |
|  | No.8 | No.12 | 10640 | 4336 | 40.7 |
|  | No.8 | No.14 | 9418 | 4541 | 48.2 |
|  | No.8 | No.27 | 7444 | 3702 | 49.7 |
|  | No.8 | No.30 | 9468 | 4309 | 45.5 |
|  | No.12 | No.14 | 22672 | 10740 | 47.4 |
|  | No.12 | No.21 | 21941 | 9216 | 42.0 |
|  | No.12 | No.25 | 20516 | 8750 | 42.6 |
|  | No.12 | No.27 | 13512 | 5536 | 41.0 |
|  | No.12 | No.30 | 20663 | 9016 | 43.6 |
|  | No.14 | No.15 | 16321 | 7997 | 49.0 |
|  | No.14 | No.21 | 18432 | 8527 | 46.3 |
|  | No.14 | No.25 | 17542 | 8341 | 47.5 |
|  | No.14 | No.27 | 12122 | 5567 | 45.9 |
|  | No.15 | No.30 | 15359 | 7227 | 47.0 |
|  | No.21 | No.8 | 9753 | 4073 | 41.8 |
|  | No.21 | No.25 | 17248 | 7321 | 42.4 |
|  | No.21 | No.27 | 12031 | 5007 | 41.6 |
|  | No.21 | No.30 | 17168 | 7540 | 43.9 |
|  | No.25 | No.8 | 9601 | 4082 | 42.5 |
|  | No.25 | No.27 | 11887 | 5202 | 43.7 |
|  | No.25 | No.30 | 16538 | 7353 | 44.5 |
|  | No.27 | No.30 | 11496 | 5244 | 45.6 |
|  | No.30 | No.2 | 9158 | 3804 | 41.5 |
|  | No.30 | No.31 | 14445 | 6882 | 47.6 |
| C1 | No.4 | No.5 | 11062 | 1644 | 14.9 |
|  | No.4 | No.9 | 13647 | 3245 | 23.8 |
|  | No.4 | No.10 | 12688 | 2329 | 18.3 |
|  | No.4 | No.22 | 14440 | 2644 | 18.3 |
|  | No.4 | No.33 | 19789 | 2330 | 11.7 |
|  | No.5 | No.9 | 11524 | 2104 | 18.2 |
|  | No.5 | No.10 | 9732 | 2091 | 21.5 |
|  | No.5 | No.22 | 10809 | 2346 | 21.7 |
|  | No.5 | No.33 | 10627 | 3710 | 34.9 |
|  | No.5 | No.43 | 13879 | 2123 | 15.3 |
|  | No.9 | No.10 | 14826 | 2419 | 16.3 |
|  | No.9 | No.22 | 17505 | 2861 | 16.3 |
|  | No.9 | No.33 | 23071 | 3229 | 14.0 |
|  | No.9 | No.43 | 23301 | 3765 | 16.1 |
|  | No.9 | No.44 | 23635 | 4470 | 18.9 |
|  | No.10 | No.22 | 10348 | 4981 | 48.1 |
|  | No.10 | No.33 | 17026 | 2845 | 16.7 |
|  | No.10 | No.43 | 18700 | 2756 | 14.7 |
|  | No.10 | No.44 | 19662 | 3046 | 15.5 |
|  | No.10 | No.48 | 10646 | 1921 | 18.0 |
|  | No.10 | No.49 | 11075 | 1978 | 17.9 |
|  | No.22 | No.33 | 21227 | 3817 | 18.0 |
|  | No.22 | No.43 | 22792 | 3530 | 15.5 |
|  | No.22 | No.44 | 24017 | 4014 | 16.7 |
|  | No.22 | No.48 | 12569 | 2321 | 18.5 |
|  | No.22 | No.49 | 12670 | 2156 | 17.0 |
|  | No.33 | No.43 | 34734 | 5058 | 14.6 |
|  | No.33 | No.44 | 41743 | 4819 | 11.5 |
|  | No.33 | No.48 | 15901 | 1708 | 10.7 |
|  | No.33 | No.49 | 16741 | 1618 | 9.7 |
|  | No.43 | No.4 | 19758 | 2748 | 13.9 |
|  | No.43 | No.44 | 39548 | 6515 | 16.5 |
|  | No.43 | No.48 | 15409 | 2257 | 14.6 |
|  | No.43 | No.49 | 15688 | 2423 | 15.4 |
|  | No.44 | No.4 | 18882 | 4128 | 21.8 |
|  | No.44 | No.5 | 16040 | 1730 | 10.8 |
|  | No.44 | No.48 | 14058 | 3184 | 22.6 |
|  | No.44 | No.49 | 15845 | 2852 | 18.0 |
|  | No.48 | No.4 | 7961 | 4030 | 50.7 |
|  | No.48 | No.5 | 9636 | 1556 | 16.1 |
|  | No.48 | No.9 | 11082 | 2773 | 25.0 |
|  | No.48 | No.49 | 9572 | 2154 | 22.5 |
|  | No.49 | No.4 | 11033 | 2402 | 21.8 |
|  | No.49 | No.5 | 9969 | 1397 | 14.0 |
| C2 | No.7 | No.11 | 13991 | 3218 | 23.0 |
|  | No.7 | No.23 | 15078 | 3307 | 21.9 |
|  | No.11 | No.23 | 15539 | 4533 | 29.2 |
|  | No.11 | No.24 | 13055 | 7026 | 53.8 |
|  | No.23 | No.24 | 16974 | 4967 | 29.3 |
|  | No.24 | No.7 | 15418 | 3432 | 22.2 |
| C3 | No.1 | No.3 | 13815 | 5548 | 40.1 |
|  | No.1 | No.16 | 20076 | 3907 | 19.5 |
|  | No.1 | No.18 | 21603 | 3617 | 16.7 |
|  | No.1 | No.20 | 20758 | 3426 | 16.5 |
|  | No.3 | No.16 | 19858 | 4018 | 20.2 |
|  | No.3 | No.18 | 21426 | 3877 | 18.1 |
|  | No.3 | No.20 | 20615 | 3629 | 17.6 |
|  | No.16 | No.18 | 26168 | 5011 | 19.1 |
|  | No.16 | No.20 | 25155 | 5146 | 20.4 |
|  | No.16 | No.37 | 16636 | 4104 | 24.7 |
|  | No.18 | No.20 | 25963 | 4660 | 17.9 |
|  | No.18 | No.37 | 18162 | 3476 | 19.1 |
|  | No.18 | No.41 | 12343 | 2167 | 17.5 |
|  | No.20 | No.37 | 16802 | 3785 | 22.5 |
|  | No.20 | No.41 | 12048 | 2111 | 17.5 |
|  | No.20 | No.45 | 14778 | 3065 | 20.7 |
|  | No.37 | No.1 | 15480 | 3017 | 19.5 |
|  | No.37 | No.3 | 15195 | 3039 | 20.0 |
|  | No.37 | No.41 | 9447 | 2057 | 21.7 |
|  | No.37 | No.45 | 11183 | 2933 | 26.2 |
|  | No.41 | No.1 | 8279 | 3471 | 29.8 |
|  | No.41 | No.3 | 8259 | 3727 | 45.1 |
|  | No.41 | No.45 | 8261 | 2129 | 25.7 |
|  | No.45 | No.1 | 13057 | 2782 | 21.3 |
|  | No.45 | No.3 | 13008 | 2948 | 22.6 |
|  | No.45 | No.16 | 14067 | 3708 | 26.3 |
|  | No.46 | No.1 | 23226 | 3658 | 15.7 |
|  | No.46 | No.3 | 22571 | 3769 | 16.7 |
|  | No.46 | No.16 | 25504 | 6161 | 24.1 |
| C4 | No.6 | No.13 | 24481 | 3843 | 15.7 |
|  | No.6 | No.17 | 16997 | 2973 | 17.5 |
|  | No.6 | No.19 | 18538 | 3001 | 16.2 |
|  | No.6 | No.28 | 20002 | 3227 | 16.1 |
|  | No.13 | No.17 | 21559 | 3719 | 17.2 |
|  | No.13 | No.19 | 23203 | 3083 | 13.3 |
|  | No.13 | No.28 | 26763 | 3199 | 11.9 |
|  | No.13 | No.29 | 25158 | 3033 | 12.0 |
|  | No.17 | No.19 | 16086 | 2446 | 15.2 |
|  | No.17 | No.28 | 17262 | 2801 | 16.2 |
|  | No.17 | No.32 | 12418 | 5361 | 43.9 |
|  | No.17 | No.38 | 6537 | 3521 | 53.9 |
|  | No.19 | No.28 | 18991 | 2819 | 14.8 |
|  | No.19 | No.29 | 18770 | 2647 | 14.1 |
|  | No.19 | No.32 | 18220 | 2795 | 15.3 |
|  | No.19 | No.38 | 20175 | 2568 | 12.7 |
|  | No.28 | No.29 | 19650 | 2949 | 15.0 |
|  | No.28 | No.32 | 19401 | 3170 | 16.3 |
|  | No.28 | No.38 | 21530 | 3022 | 14.0 |
|  | No.28 | No.40 | 24172 | 3516 | 14.5 |
|  | No.29 | No.38 | 20526 | 2926 | 14.2 |
|  | No.29 | No.40 | 22886 | 3004 | 13.1 |
|  | No.29 | No.47 | 3007 | 131 | 4.3 |
|  | No.29 | No.50 | 13771 | 1579 | 11.4 |
|  | No.32 | No.38 | 21199 | 2841 | 13.4 |
|  | No.32 | No.40 | 22594 | 3898 | 17.2 |
|  | No.32 | No.47 | 3104 | 134 | 4.3 |
|  | No.38 | No.6 | 21640 | 2877 | 13.3 |
|  | No.38 | No.40 | 26045 | 3230 | 12.4 |
|  | No.38 | No.47 | 3116 | 131 | 4.2 |
|  | No.40 | No.6 | 22133 | 4483 | 20.2 |
|  | No.40 | No.13 | 31598 | 4869 | 15.4 |
|  | No.40 | No.47 | 3342 | 118 | 3.5 |
|  | No.47 | No.6 | 2473 | 136 | 5.4 |
|  | No.47 | No.13 | 2874 | 105 | 3.6 |
|  | No.47 | No.17 | 2397 | 110 | 4.5 |
|  | No.50 | No.6 | 12063 | 2425 | 20.1 |
|  | No.50 | No.13 | 15576 | 1879 | 12.1 |
|  | No.50 | No.17 | 11946 | 1823 | 15.3 |
|  | No.50 | No.19 | 12909 | 1838 | 14.2 |

**Supplementary Fig. S1.** Principal component analysis (PCA) of *Allium macrostemon* used in this study. The five major component data are shown by the five two-dimensional data sets: (**a**) first and third PCA axes, (**b**) first and fourth PCA axes, and (**c**) first and fifth PCA axes.


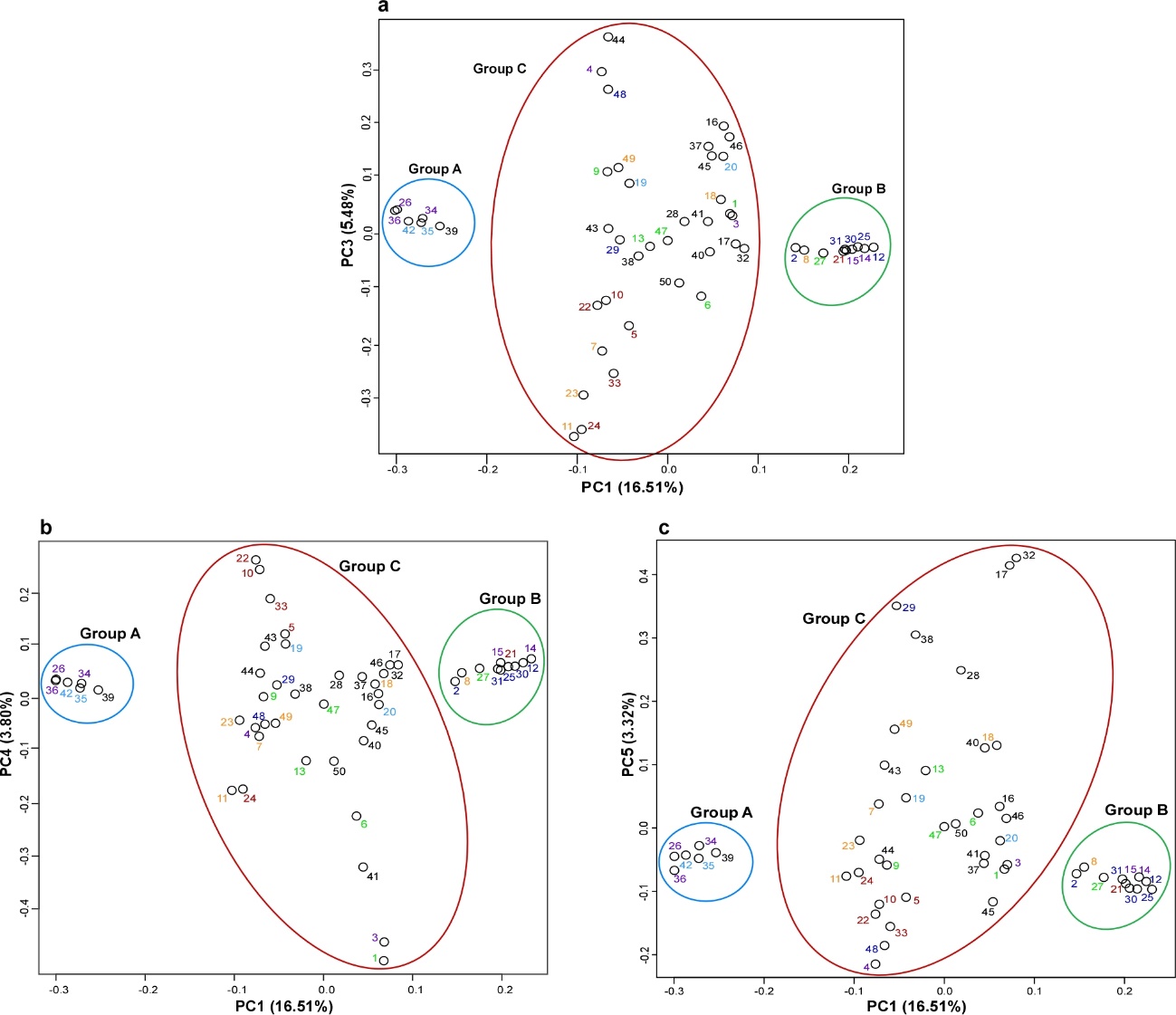


**Supplementary Fig. S2.** Cross-validation (cv) error plotted against the number of ancestors (*K*) at *K*=1-10 and *K*= 1,6,7,8.9,10 admixture plots.


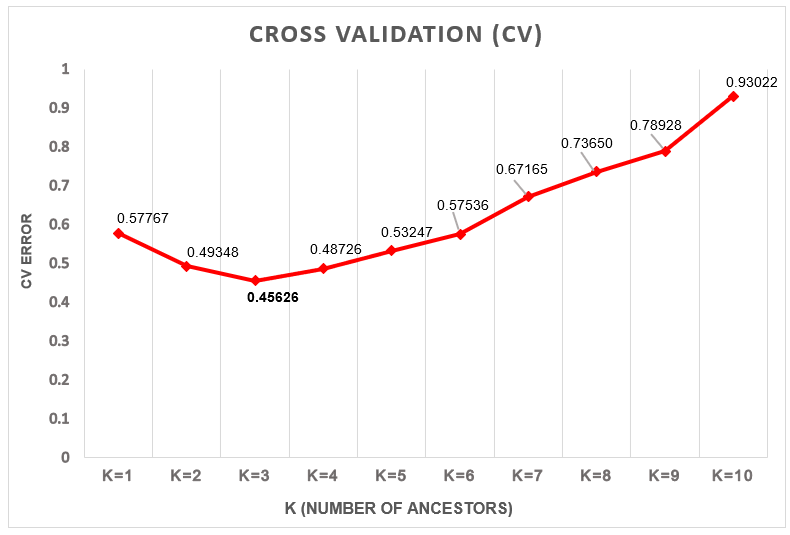


**Supplementary Fig. S3.** *K*= 1, *K*= 6, *K*= 7, *K*= 8, *K*= 9, *K*= 10 admixture plots in admixture analysis of *Allium macrostemon* individuals used in this study. The horizontal axis shows the group names and the sample numbers.


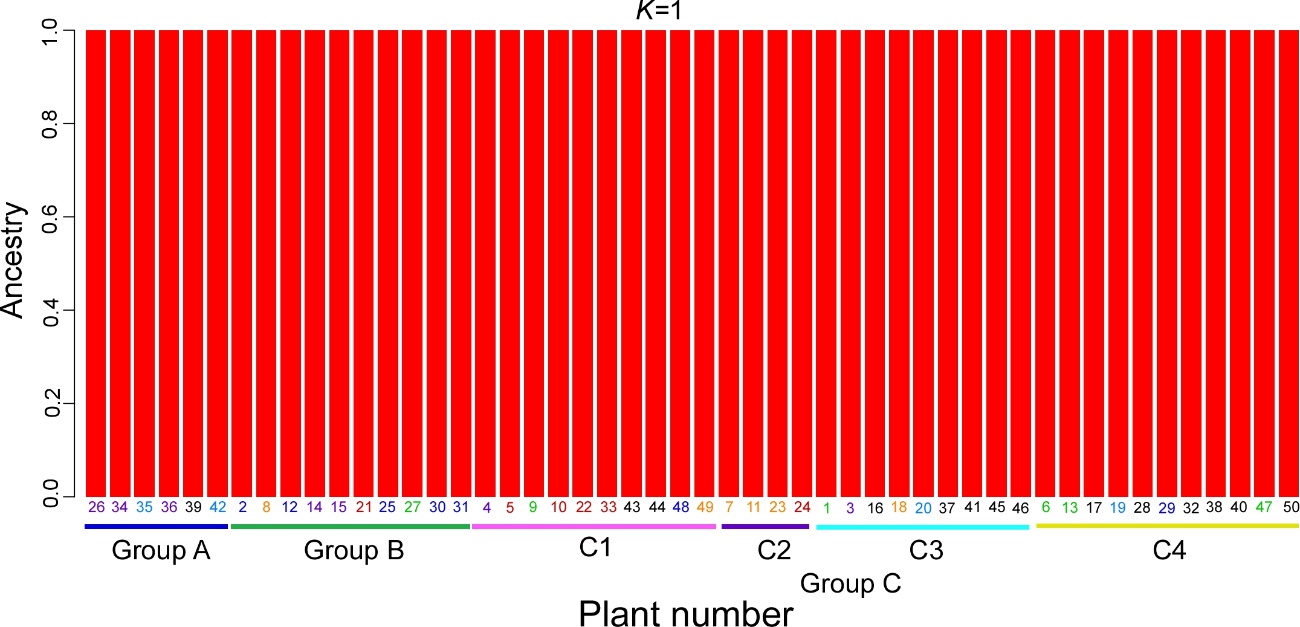


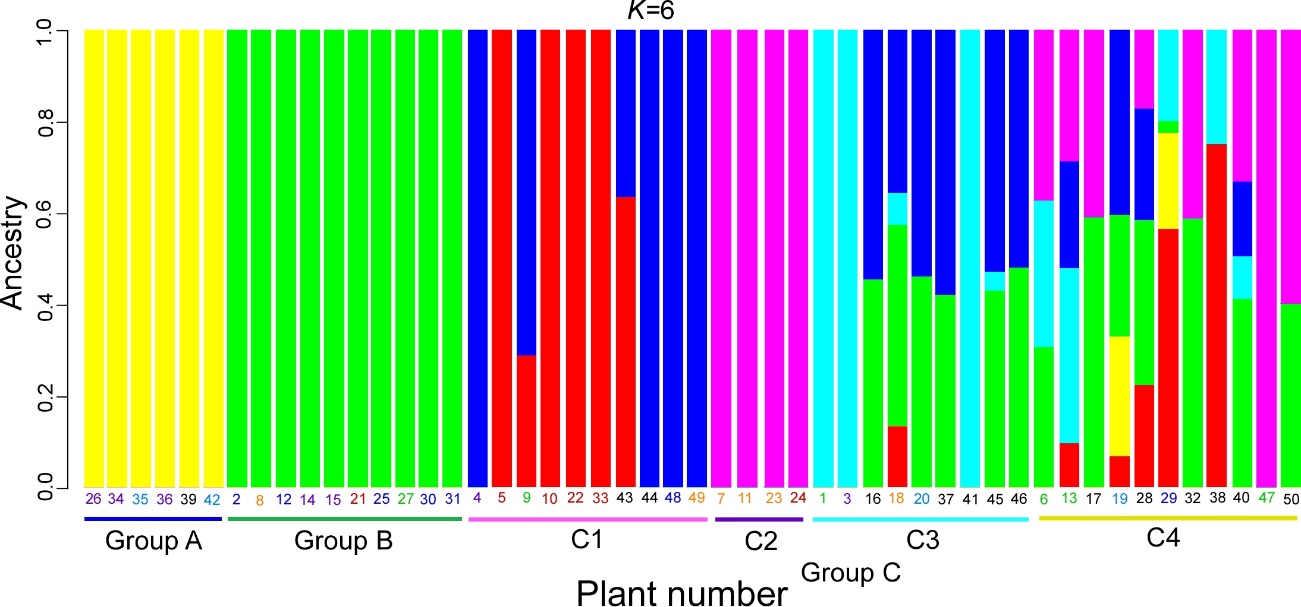


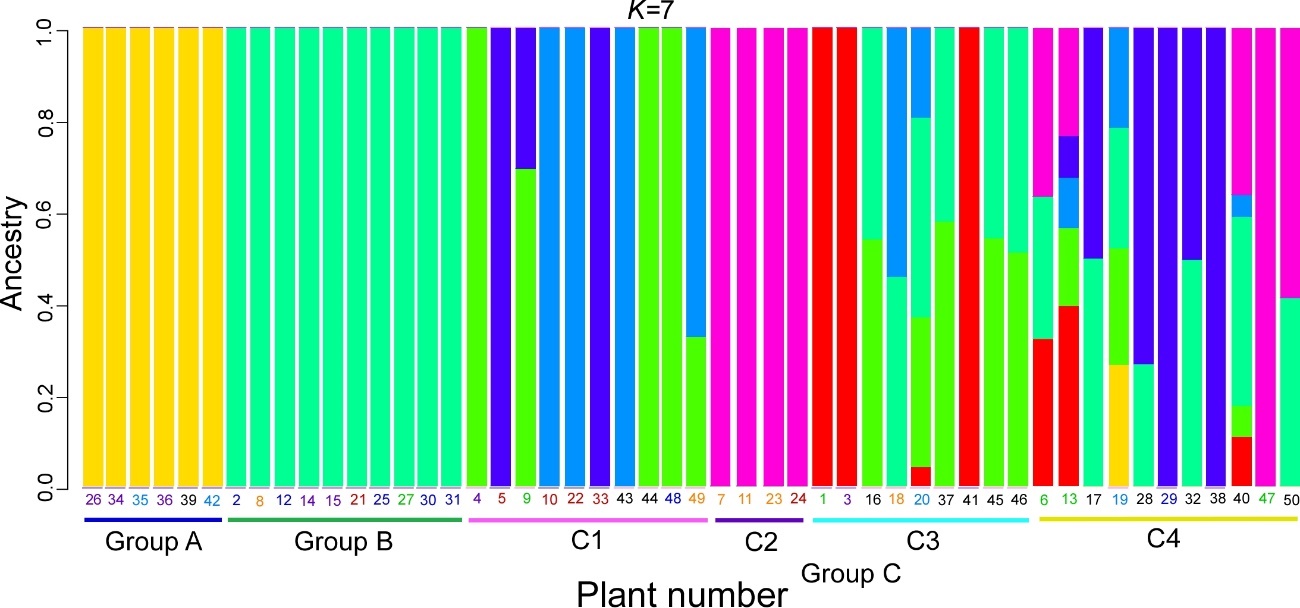


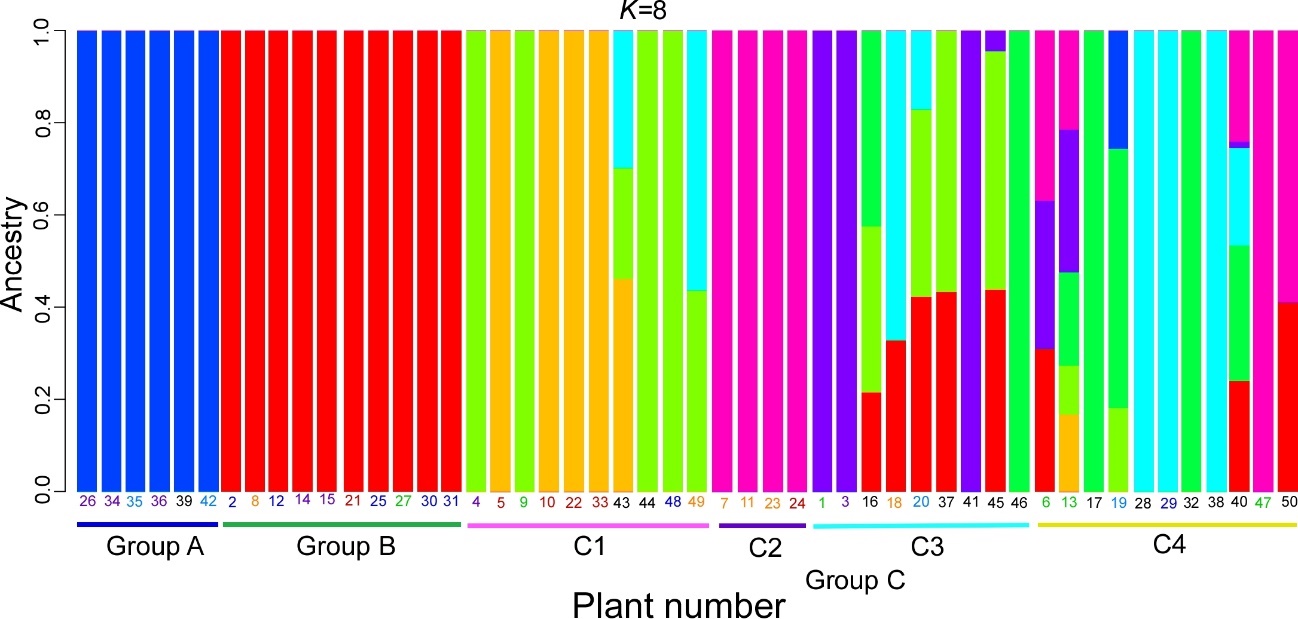


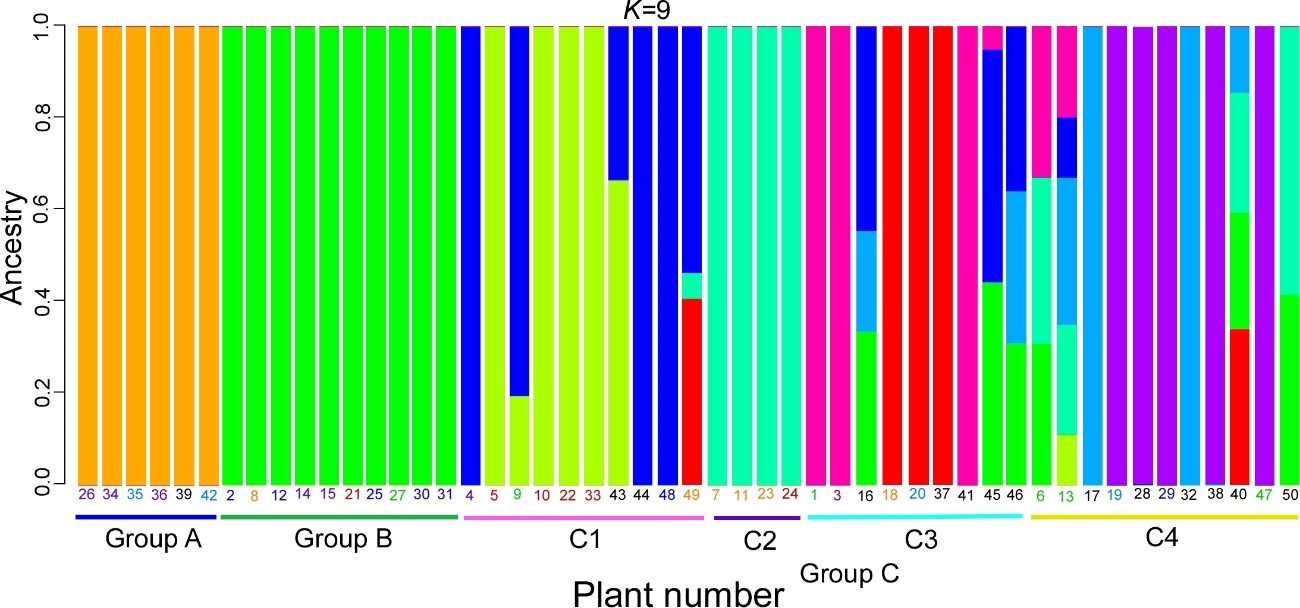


| 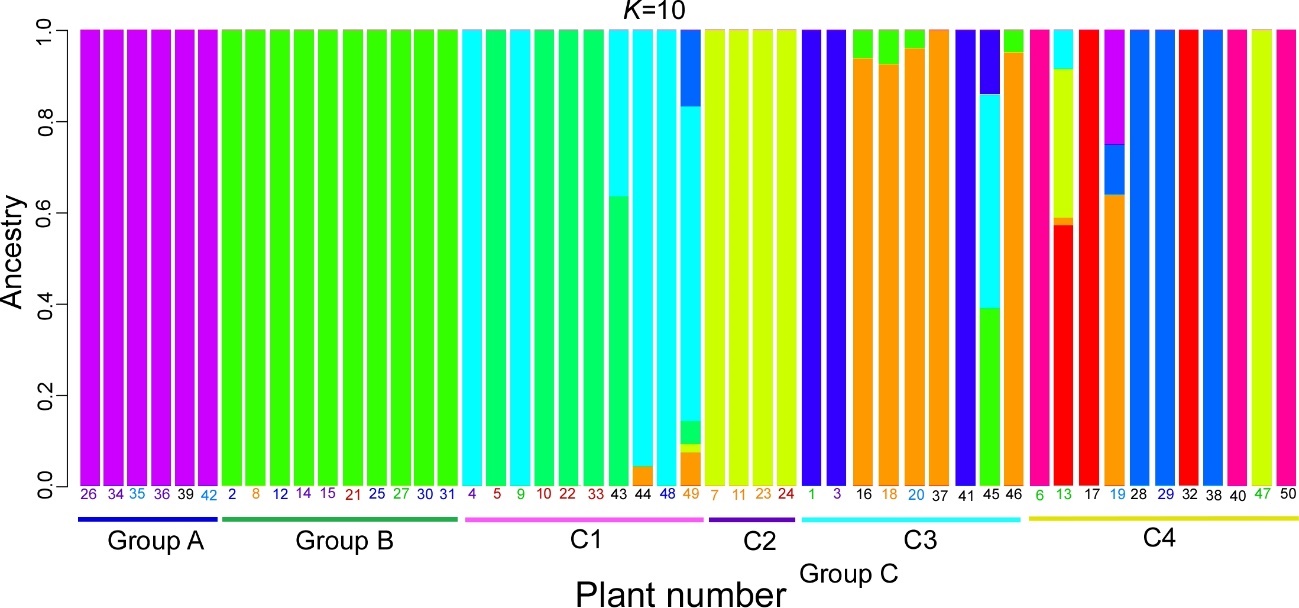 |
| --- |
